# Programmable photonic neural engine with all-optical nonlinear activation and 40,000 connections

**DOI:** 10.1126/sciadv.aee9649

**Published:** 2026-08-19

**Authors:** Hao Sun, Xinyi Zhu, José Azaña

**Affiliations:** Institut National de la Recherche Scientifique, Centre Énergie Matériaux Telecommunications (INRS-EMT), Montréal, QC H5A-1K6, Canada.

## Abstract

The rapid advancement of artificial neural networks (ANNs) demands computational platforms with higher speed, energy efficiency, and scalability. Optical computing offers an appealing solution owing to the high-speed processing, inherent parallelism, and low energy consumption. However, existing optical neural network architectures face fundamental trade-offs between scalability, reconfigurability, and processing latency, constrained by the need to rely on electronic nonlinearities or fixed optical interconnections. Here, we report an end-to-end photonic neuromorphic engine that integrates all-optical nonlinearity into a loop-based, time-multiplexed photonic architecture. This approach enables all optical neurons to share common hardware, supporting enhanced connectivity and full reconfigurability with reduced latency. Implementations of four distinct network topologies, with a maximum of 40,000 optical connections for the single-layer perceptron, achieve digital-level inference accuracy and computational latency over two orders of magnitude shorter than state-of-the-art electronic processors, potentially paving the way for scalable, reconfigurable, and ultrafast optical artificial intelligence systems.

## INTRODUCTION

Artificial neural networks (ANNs), particularly deep neural networks, are based on large-scale connections of individual neuron elements, each involving linear multiply-accumulate (MAC) operations followed by a nonlinear activation function (NLAF), extending both in depth (comprising many layers of neurons) and in width (comprising many neurons per layer) (*1*). State-of-the-art ANNs are implemented through digital electronics, allowing versatile reconfiguration and optimization of the network topology for target tasks. However, their ever-growing complexity substantially increases computational demands, pushing electronic processors to their physical and energy-efficiency limits. More importantly, the resulting computational latency becomes a critical bottleneck for applications that require real-time decision-making and interaction, such as high-speed communications (*2*, *3*), self-driving (*4*), and ultrafast optical sensing (*5*). These challenges have prompted growing interest in alternative computing paradigms (*6*–*9*).

Among these, optical neural networks (ONNs) have emerged as a promising solution to implement ANN-based tasks with ultrahigh throughput, ultralow latency, and intrinsic energy-efficiency (*9*–*22*). Most existing ONNs perform MAC operations through ultrafast and efficient linear optics. However, NLAF units and the inter-layer data transmission still heavily rely on power-inefficient and time-consuming digital or analog electronics (*9*–*17*). This requirement increases the processing latency along the network, hindering the speed advantage of the optical approach. End-to-end ONN architectures can readily accelerate the overall neural operations by physically connecting adjacent neural layers through optical waveguides. Yet, in most schemes, optoelectronic (OE) and electro-optic (EO) conversions are still required to implement the NLAF units (*18*–*20*). This requirement represents a crucial hurdle for scaling up the number of neurons and/or layers. All these limitations are exacerbated by the stacked architectures used in current end-to-end ONNs (*18*–*20*). As networks scale in depth and width, insertion losses accumulate and system complexity grows rapidly, ultimately imposing substantial additional constraints on scalability. To the best of our knowledge, end-to-end ONNs demonstrated to date remain small in scale, supporting less than a few tens of neurons or connections and a restricted fan-in per neuron (tens of input features at most) (*18*–*20*).

To avoid the inefficient OE and EO conversions, all-optical neural networks (AONNs), incorporating all-optical NLAFs, have been envisioned towards a full realization of the potential of ONNs (*21*–*23*). However, demonstrated AONN architectures support only up to a few tens of neurons. This limitation arises primarily from the stacked architectures used (*21*–*23*) and the need for a power-intensive nonlinear optical activation function at each neuron, often relying on exotic materials or complex mechanisms (*21*, *22*).

Another critical constraint of ONNs is their extremely limited reconfigurability. In contrast to digital solutions, current end-to-end ONNs are typically implemented with fixed, hardware-embedded interconnection architectures (*18*–*20*). In such systems, both neuron functionality and connections are physically defined, making modifications to network depth or width difficult and often requiring substantial changes to the underlying photonic hardware, such as adding or removing optical components. Consequently, adapting a given ONN to different network topologies generally requires hardware redesign rather than simple reprogramming. This lack of tunability markedly limits the practical deployment of ONNs for general-purpose neural computing. Therefore, developing a scalable and reconfigurable end-to-end ONN architecture, comprising efficient all-optical implementation of the NLAF units and neural connections, remains challenging.

**Fig. 1.**
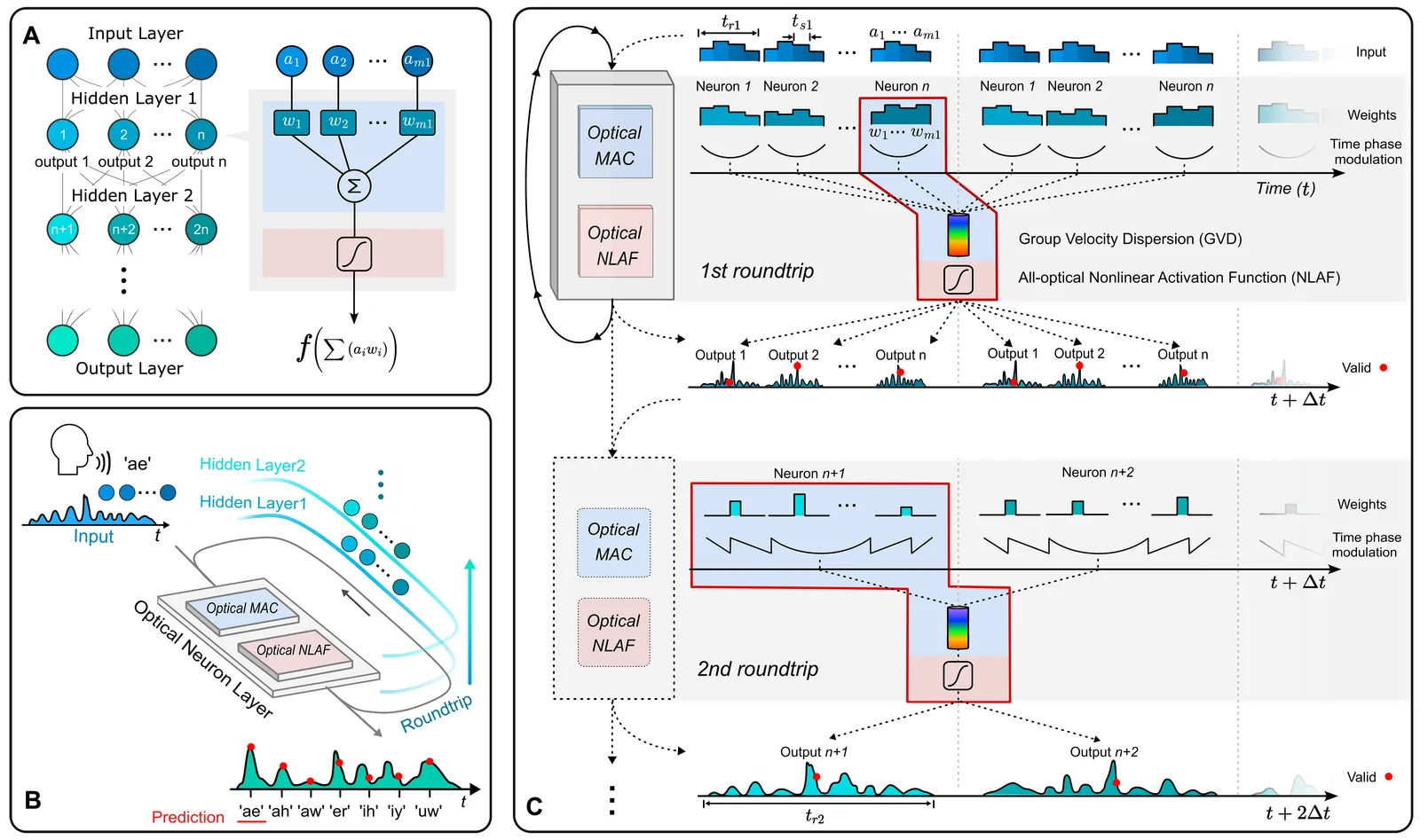
Conceptual illustration of the end-to-end ONN. (**A**) DNN and a single neuron. The multiply-accumulate (MAC) operation of the neuron involves (i) elementwise multiplication between inputs ($a_{i=1,2,\ldots m_{1}}$) and weights ($w_{i=1,2,\ldots ,m_{1}}$) and (ii) coherent accumulation of the resulting products, while the nonlinearity (f) rescale the summation result. Note that $m_{1}$ represents the fan-in of the first layer. (**B**) The overview of the loop-based, time-multiplexed end-to-end ONN. Neuron layers are folded consecutively into the corresponding roundtrips, e.g., the neurons in the first layer are time-encoded in the first roundtrip and so on. In the example task, the input features of the vowel ‘ae’ are fed into the ONN. After several roundtrips (layers), the network provides seven different outputs encoded along time (valid outputs: red dots), corresponding to the different labels to be discerned, from ‘ae’ to ‘uw’. The maximum output value represents the prediction. (**C**) Conceptual illustration of the optical neurons and ONN. As an example, n neurons in the first layer are presented, while two neurons are shown for the second layer. Two single neurons are highlighted by red boxes. Experimental implementations are detailed in Materials and Methods.

Here, we propose and experimentally demonstrate an ONN scheme enabling an enhanced number of connections, low processing latencies, and versatile reconfigurability for user-defined implementation of different ANN topologies. Unlike conventional stacked architectures, the proposed design is an optical loop-based, time-multiplexed structure where all neurons and layers share the same photonic hardware. Specifically, a single optical linear operation unit and a single all-optical nonlinear element are used to perform all neural operations (Fig. 1, A and B), dramatically simplifying the required hardware. As each roundtrip through the loop corresponds to one neural layer, the per-layer propagation loss remains constant, regardless of the number of neurons per layer. Additionally, optical MAC operations are realized via stable temporal interference, supporting a large fan-in per neuron. In proof-of-concept experiments, we first demonstrate a single-layer perceptron (SLP) with 40,000 connections (200 neurons × 200 input features), successfully classifying 200 random binary patterns. Because neurons are time-encoded, the network structure and functionality can be easily reprogrammed, without modifying the physical configuration. We demonstrate here various well-established distinct ANN topologies using the same photonic hardware, from shallow networks (the aforementioned SLP and a two-layer CNN for vowels recognition) to deeper models (a four-layer DNN for solving the logic exclusive-or problem and a three-layer DNN for iris plants’ classification). All networks are trained using an effective physics-aware training (PAT) method specifically optimized for our architecture (*9*, *24*), achieving accuracies on par with digital neural networks and with computational latencies orders of magnitude shorter than those of a state-of-art electronic processor.

## RESULTS

### Principle

One representative ANN topology is the multilayer perceptron (MLP), constructed by stacking multiple neuron layers (Fig. 1A). To implement this network in a highly efficient manner, each layer is projected into a single roundtrip through a photonic processor, while the neurons per layer are arranged consecutively along the time domain (Fig. 1B). After a single roundtrip, the outputs of the neuron layer serve as the inputs of the next one by recirculating the resulting optical waveforms through the photonic processor.

Figure 1C illustrates the detailed implementation of the loop-based ONN. To realize a single neuron in the first layer (highlighted with a red box), two temporal modulation units are utilized to respectively encode the $m_{1}$ input features ($a_{1},a_{2},\ldots ,a_{m1}$) and the $m_{1}$ weights ($w_{1},w_{2},\ldots ,w_{m1}$) into the optical domain. Specifically, the input features are mapped onto an optical waveform through time-dependent amplitude modulation, where each feature value is assigned to a temporal slot of duration $t_{s1}$. In practice, the modulators are biased at $V_{\pi }$, such that the modulation induces a signed scaling of the optical electric field, enabling the representation of both positive and negative values. The weights are encoded in the same manner by a second synchronized modulation unit.

As the optical signal propagates through the two cascaded modulation units, each modulator independently scales the optical field. As a result, the optical field in each temporal slot becomes proportional to the product of the corresponding input feature and weight, thereby implementing the target elementwise multiplication process (*25*–*27*). Each encoded feature–weight pair occupies a temporal duration of $t_{s1}$, resulting in a time-multiplexed product vector extending over $t_{r1}=m_{1}t_{s1}$. The time period $t_{r1}$ determines the temporal aperture parameter for the following photonic accumulation process. Detailed descriptions regarding the temporal aperture concept are provided in the Supplementary Text, section S1.

The accumulation of the elements of the resulting product vector are accomplished using the focusing principle of a time lens (*28*). For this purpose, a Fresnel time lens (FTL) (*28*–*30*) applies a quadratic temporal phase modulation ($\varphi_{0}t^{2}/2$) on the optical product vector over the corresponding period $t_{r1}$, followed by a group-velocity dispersion (GVD) component. When the dispersion coefficient $\ddot{\phi }$ of the GVD unit (slope of the delay shift as a function of frequency) precisely compensates for the frequency chirp introduced by the FTL, namely, $-1/\ddot{\phi }=\varphi_{0}$, the consecutive data along the lens temporal aperture, i.e., the elements of the product vector, are synchronized and coherently accumulated at the time-lens focal point, located at the center of the output time slot corresponding to the lens aperture (Supplementary Text, section S1). In particular, the Fresnel temporal phase profile is constructed by wrapping the target quadratic phase modulation waveform within the range [−π,π); this is a key strategy to achieve a massive fan-in and highly scalable DNNs (Materials and Methods). Subsequently, the linear summation output is sent to an all-optical nonlinear element for implementation of the NLAF, accomplishing the functionality of a single neuron. Multiple neurons can then be realized by consecutively encoding the corresponding input features and weights into two synchronized, time-multiplexed sequences, with the feature/weight vector corresponding to each neuron extending over a period $t_{r1}$. As such, the first roundtrip through the photonic processor can effectively implement n different neurons, yielding the desired n valid outputs (red dots in Fig. 1C) consecutively along the time domain. This is achieved using a single set of optical linear and nonlinear processing units.

In the second roundtrip/layer, the n outputs from the first layer (n first outputs from the left in Fig. 1C) are recirculated into the photonic processor using an optical path (e.g., the standard optical fiber utilized in our experiments), serving as the inputs of the “neuron n+1”. The weights of “neuron n+1” are now designed as rectangular pulses to filter out unnecessary information while keeping the valid outputs only. To optimize the energy efficiency, the pulse width is set to $t_{s1}$, the temporal duration per feature in the first layer (Supplementary Text, section S1). The temporal aperture ($t_{r2}$) per neuron of the second layer is then given by $nt_{r1}$. To perform the accumulation process using the same GVD element, the time lens must be designed to impose an identical frequency chirp on the incoming light wave though now extending over a longer temporal aperture ($t_{r2}$). Hence, similarly to the operation in the first layer, the outputs of the (n+1)-th neuron, and those of subsequent neurons in the second layer, are also consecutively obtained along the time domain through the same optical hardware. Obviously, for implementation of k neurons in the second layer (for simplicity, k=n in Fig. 1A), the temporal structures corresponding to the n neurons in the first layer should be replicated by k times in the first roundtrip (Fig. 1C). The same process is then repeated to implement all subsequent layers in the designed ANN.

In the work reported here, the parameters of the neurons are encoded into the optical domain using electro-optic modulators driven by an arbitrary waveform generator (AWG) (Materials and Methods). This enables straightforward electronic reconfigurability to program different ANN topologies, including distinct architectures such as CNNs and MLPs, and their corresponding parameters (number of input features, neurons and layers, and value of weights), while the neuromorphic computing is efficiently performed in the optical domain. This parameter reconfigurability is also essential for effective implementation of the PAT.

### All-optical nonlinear activation function

**Fig. 2.**
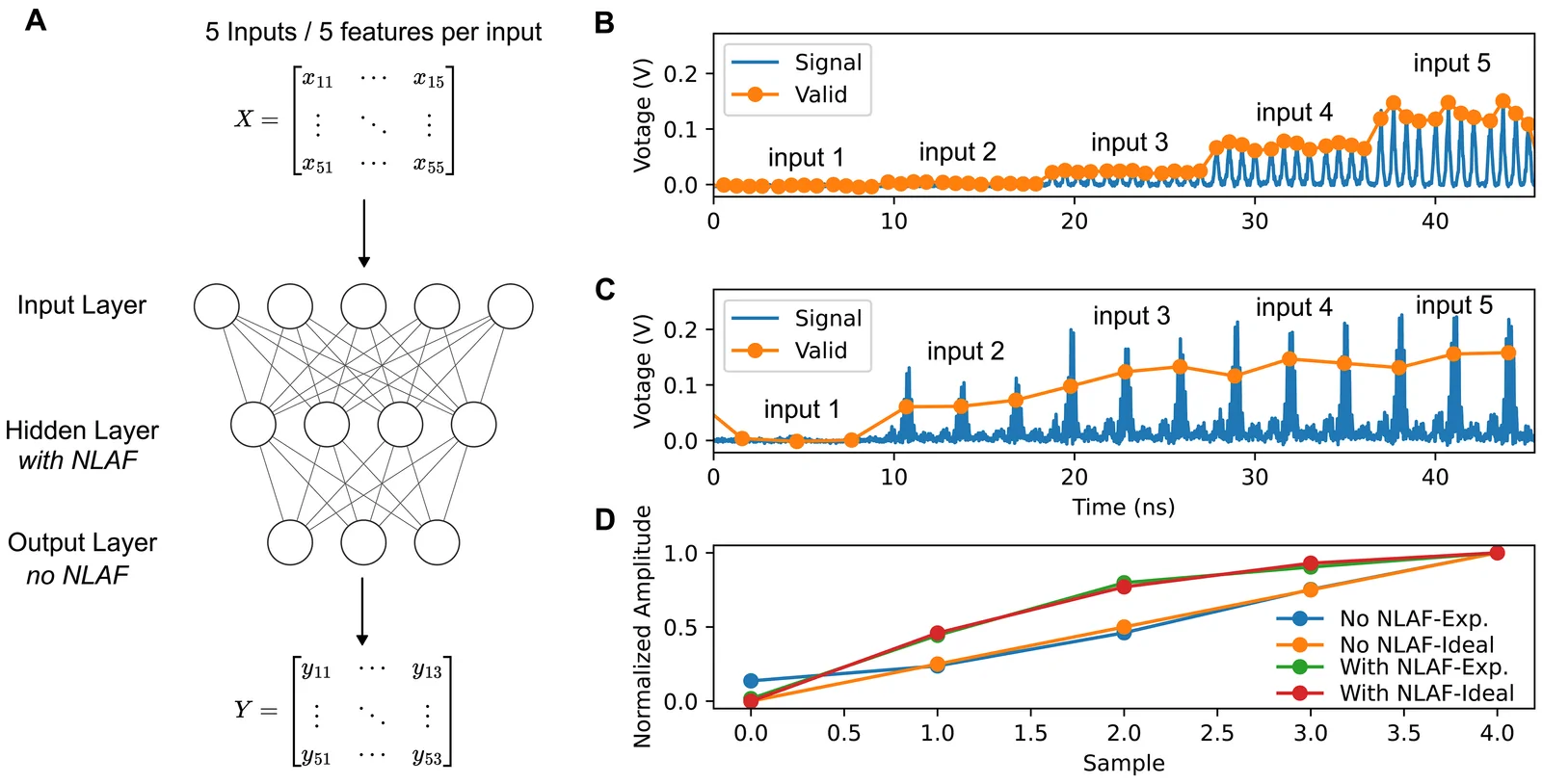
Calibration of the all-optical nonlinear activation function to implement a *tanh* thresholding function. (**A**), Architecture of the neural network for calibration of the NLAF. (**B**), Experimental output (optical power) of the hidden layer before the NLAF unit (SOA). (**C**), Experimental output (optical power) of the final output layer. The time slots of input samples are highlighted in (B) and (C). (**D**), Comparison between the experimental and ideal *tanh* responses.

A commercially available semiconductor optical amplifier (SOA) is here utilized as the NLAF component. This is suitably operated to realize a hyperbolic tangent (*tanh*) NLAF (*31*), which is widely utilized in ANNs (*1*). To calibrate the optical NLAF, we design a two-layer MLP, as shown in Fig. 2A. The 5 inputs’ samples (row in matrix **X**), each with 5 features, will result in a 5 × 3 output matrix **Y**, where each row corresponds to the output of one input sample. Specifically, each output/row ($Y_{i}=\left[y_{i1},y_{i2},y_{i3}\right]$) is designed to have the same values ($y_{i1}=y_{i2}=y_{i3}$), and we engineer the input matrix and weights to obtain increased values across the 5 consecutive outputs. The corresponding temporal waveforms of the hidden layer and output layer are shown in Fig. 2 (B and C), respectively. The SOA can then be calibrated by comparing the normalized experimental outputs with the normalized ideal results. When the current of the SOA is set to ∼130 mA, the extracted response of the SOA is in excellent agreement with the response of the ideal *tanh* function, see Fig. 2D. More details in this calibration procedure are presented in the Materials and Methods.

### Large-scale single-layer perceptron

**Fig. 3.**
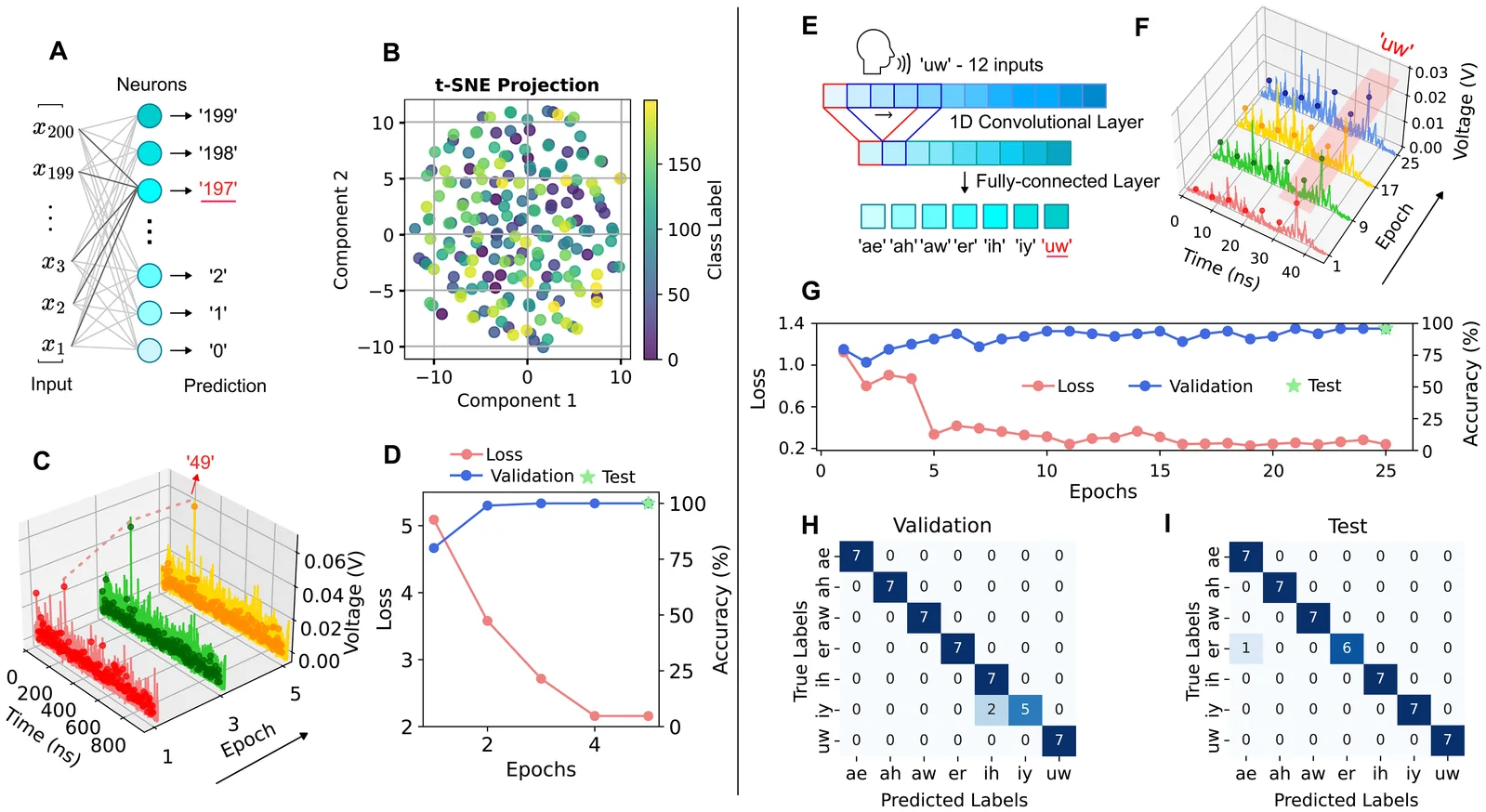
Experimental results of shallow ONNs. (**A**) 200 neurons in one layer for recognition of 200 different binary patterns; the vector to be classified consists of 200 bits, which are the input features of each optical neuron. (**B**) t-SNE projection of the 200 binary patterns. (**C**) Output waveforms enabling the prediction of the input of class 49. Specifically, there are 200 periods, each extending over ∼4.5 ns, corresponding to the 200 neurons. (**D**) The validation accuracy and loss during training. (**E**) One-dimensional (1D) convolutional neural network (CNN) for the classification of 7 vowels, including one convolutional layer and one fully connected (FC) layer. (**F**) Output signals of the FC layer for different training epochs. There are seven periods each extending over ∼5.4 ns, corresponding to the seven vowels. In this example, the seventh valid output attains the highest value, corresponding to the maximum likelihood for the vowel ‘uw’. (**G**) The validation accuracy and loss as a function of training epochs. (**H**) and (**I**), The confusion matrices of the validation and test datasets, respectively.

We first implement a large-scale single-layer perceptron (SLP) for one-dimensional (1D) binary pattern classification, a task relevant to optical header recognition in high-speed optical packet-switched networks (*2*, *3*). The architecture comprises 200 time-multiplexed optical neurons, each receiving 200 input features, ultimately enabling 40,000 connections. This configuration is then trained to classify 200 binary patterns, each with 200 bits (Fig. 3A), thus demonstrating unprecedented fan-in capacity. Binary input patterns are randomly generated and broadcast to all neurons, with the predicted class assigned to the neuron yielding the maximum output amplitude. The 200-dimensional input space is projected onto a two-dimensional plane using t-distributed stochastic neighbor embedding (t-SNE) to visualize the pattern distribution (Fig. 3B) (*32*).

Training is performed using the PAT algorithm that directly tunes the weights of the optical neurons to their optimal values (Supplementary Text, section S2 for all ONNs) (*9*, *24*). Output waveforms for the 50th validation sample (class 49) are recorded across training epochs. Training is terminated after five epochs as the validation accuracy converges to 100%, while the test accuracy is 100% (Fig. 3D), comparable with its digital counterpart (see accuracies of digital models in Supplementary Text, section S3). An example of real-time classification of the test dataset is shown in movie S1.

### One-dimensional convolutional neural network

The system is then reconfigured to realize a one-dimensional (1D) CNN, evaluated on a standard seven-class vowel recognition task (*33*) (Fig. 3E). The CNN comprises one convolutional layer followed by one fully connected (FC) layer, implemented over two optical roundtrips through the photonic processing engine (Fig. 3E). In the first roundtrip, the MAC operation unit is reprogrammed to perform convolutions between inputs and kernels. Specifically, twelve input features and the convolutional kernel with four weights are rearranged along the time domain to implement the target temporal convolution, yielding nine outputs that are then fed into an all-optical NLAF (SOA) (Materials and Methods). In the second roundtrip, the weights of the FC layer are time-multiplexed following the principle described in Fig. 1C. The convolutional layer consists of nine nonlinear neurons, while the FC layer comprises seven linear neurons (without NLAFs) (*34*). Figure 3F shows the measured output waveforms for the vowel “uw” across the training epochs. The seventh output channel exhibits the maximum amplitude, indicating a correct classification. The validation accuracy converges to ∼96% at the 25th epoch, while the test accuracy reaches ∼98% (Fig. 3, G to I). These results are on par with those achieved with digital CNNs (Supplementary Text, section S3).

### Deep neural networks

**Fig. 4.**
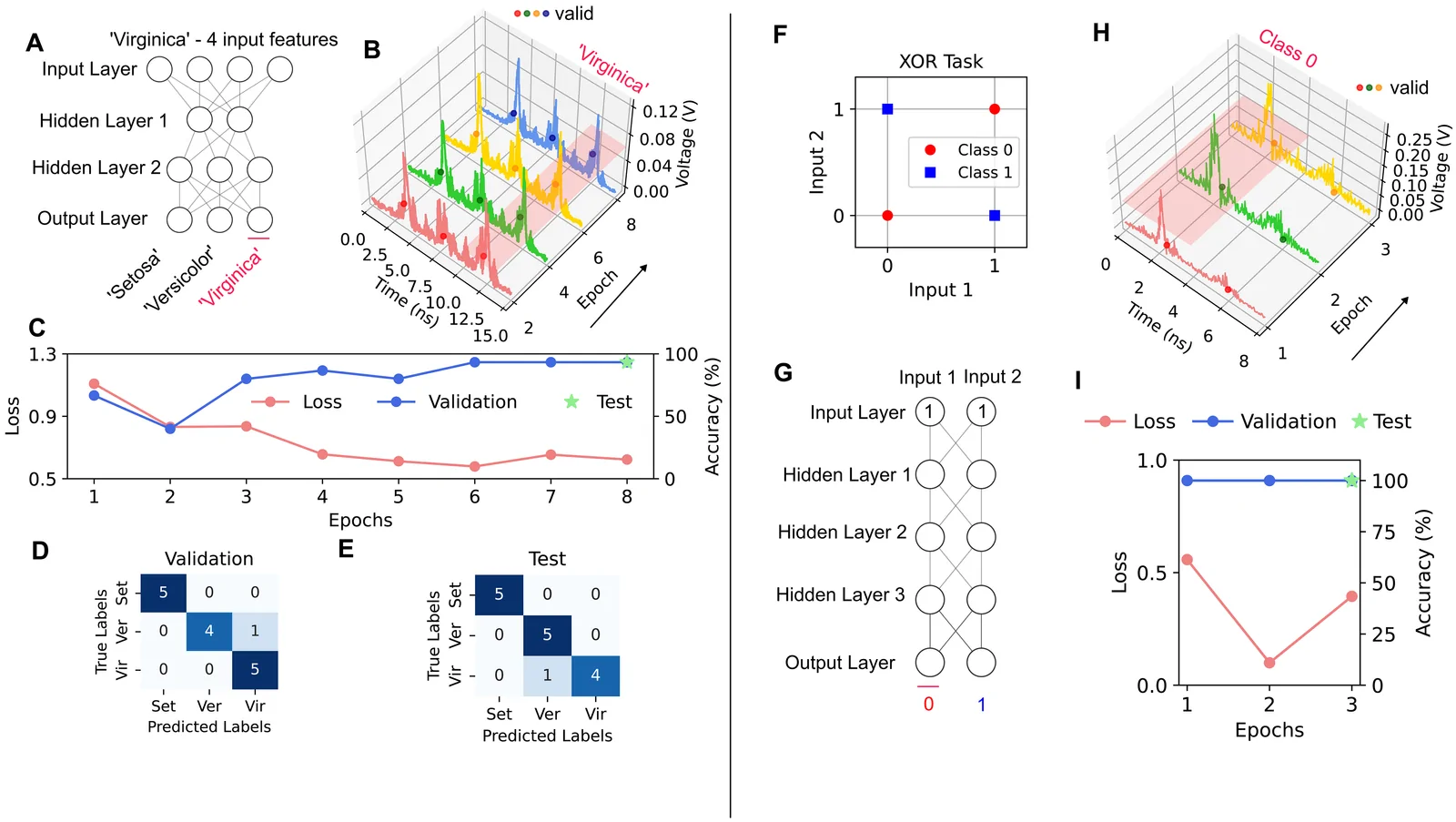
Experimental results of DNNs. (**A**) Three-layer DNN for recognition of iris plants. (**B**) The output waveforms from the output layer for the same input sample in the validation dataset (‘Virginica’). Three periods, each with around 4.2 ns, represent the three iris plant species, specifically, the first, second, and third periods correspond to ‘Setosa’, ‘Versicolor’, and ‘Virginica’, respectively. (**C**) The validation accuracy and test accuracy as a function of epochs. Both accuracies are ∼93%. (**D**) and (**E**) The confusion matrices of the validation and test dataset. (**F**) XOR problem. (**G**) Four-layer DNN for XOR logic computation. (**H**) Output waveforms from the output layer over different epochs, the input is [1, 1] for this example. (**I**) Validation and test accuracies (100%).

We further demonstrate deep neural networks (DNNs) with multiple hidden layers, which enable hierarchical feature extraction and nonlinear classification (*1*). A three-layer DNN is first implemented for classification of iris plant species (*35*) (Fig. 4A). Four input features are connected to two neurons in the first hidden layer (first roundtrip), whose outputs are then sent to three neurons in the second hidden layer (second roundtrip), followed by an output layer with three linear neurons (third roundtrip). The class label is predicted from the neuron with the highest output amplitude. The validation accuracy converges to ∼93% after eight epochs, with the test accuracy also reaching ∼93% (Fig. 4, C to E), comparable to the digital baseline (Supplementary Text, section S3). To further prove the robustness of the training pipeline and programmability of the ONN scheme, a two-layer MLP is trained by 3 times using the Iris dataset and a batch size of 10 (*36*, *37*), showcasing a comparable average test accuracy (∼97%) with its digital counterpart (see Supplementary Text, section S2.2).

To evaluate nonlinear inference capability, the ONN is reconfigured into a four-layer DNN to solve the exclusive-or (XOR) problem (Fig. 4F), a standard benchmark unsolvable by an SLP (*38*). The architecture consists of four layers, each with two neurons (Fig. 4G). Binary inputs are encoded into temporal waveforms, and classification is again based on the neuron with maximum output. For instance, input [1, 1] produces maximum amplitude from the first output neuron, corresponding to class 0. Figure 3H shows temporal output waveforms for this case, with the two periods representing outputs of the two output neurons. Training converges after only three epochs, achieving 100% accuracy on both validation and test sets (Fig. 4I), in perfect agreement with the digital implementation (Supplementary Text, section S3). The experimental implementation of all the reported ONNs is detailed in Materials and Methods. The estimated signal-to-noise ratio (SNR) of the output waveform is ∼29.31 dB (Supplementary Text, section S4), corresponding to an effective number of bits (ENOB) of ∼4.58 bits (*39*), which exceeds the commonly reported minimum requirement of ∼4 bits for performing AI tasks (*26*).

## DISCUSSION

One of the key specifications of an ANN is the computational latency, which determines the processing speed. In our experiments, the latency is mainly limited by the fiber loop roundtrip time (∼400 ns), setting the delay per layer (see Materials and Methods). Despite this non-optimized setup, the achieved latencies are in the (sub-)microsecond range, about two orders of magnitude lower than a state-of-the-art electronic processor (Supplementary Text, section S5). To optimize the latency, the long fiber loop and fiber-optics devices could be potentially integrated using compact components (Supplementary Text, section S6) (*40*–*44*). In this case, the best possible performance is determined by the temporal waveform duration per layer (Supplementary Text, section S5).

Although the latency could be improved physically by using more compact devices, the fundamental limitation is due to the need to replicate the input signals in the time domain, which, in turn, constrains the scalability of the pure time-multiplexed ONN scheme. As detailed in Supplementary Text, section S1.2, the scalability is dependent on the longest temporal aperture allowed by the GVD and modulation speed. When scaling the ONN in depth (up to 8 layers) using a commercially available linearly chirped fiber Bragg grating (LCFBG) (GVD ∼250 ns/nm) (*45*) and a 256 GS/s AWG (*46*), the width of the network becomes narrower (2 neurons per layer), and the signal duration increases consequentially (∼250 ns, Supplementary Text, section S7). In contrast, versatile fan-out approaches could enable parallel processing with high throughput and ultralow latency, but they are known to suffer from increasing insertion loss and system complexity (*13*, *14*, *18*–*20*, *23*). To address these constraints, the proposed time-multiplexed architecture could be extended by combining it with spatial-domain multiplexing (SDM), forming a hybrid time–space multiplexing framework (Supplementary Text, section S7.3), where spatial fan-out enables parallel MAC operations This could notably reduce temporal repetitions. Overall, combining time-domain processing with spatial multiplexing provides a promising route toward scalable and high-speed ONNs. Further improvements could also be potentially achieved by incorporating additional parallelization strategies such as wavelength-division multiplexing (WDM) (*10*, *14*).

Although the proposed scheme has a higher estimated energy consumption compared to existing end-to-end ONN schemes due to the longer processing latency (*18*–*20*), the overall estimated energy consumption and processing latency remains approximately 3 and 2 orders of magnitude lower than those of the NVIDIA A100 GPU, respectively (Supplementary Text, sections S5 and S8). These results highlight the relatively high energy efficiency and fast processing speed of the proposed system, together with its high-level programmability.

To realize versatile NLAFs on demand, such as the well-established ReLU or Sigmoid, suitable all-optical nonlinear units, including reconfigurable elements, could be readily incorporated into our scheme without modifying the rest of the network architecture (*47*–*49*). This emphasizes further the high level of reconfigurability offered by the proposed architecture, not only in programing ANN topologies but also in customizing the NLAF according to the target task. Although demonstrations shown here focus on feedforward neural networks, other network topologies, such as recurrent neural networks, long short-term memory, and transformer (*50*), could in principle be realized using the proposed loop-based ONN scheme. In such architectures, recurrent connections require coherent interference between the incoming input and the recirculated output waveforms from the previous layer (*50*). As such, to ensure stable interference in a fiber-based setup, active stabilization techniques such as the Pound–Drever–Hall scheme could be employed to overcome the effect of phase fluctuations in the loop (*51*). Alternatively, full photonic integration of the computing engine could further mitigate undesired phase variations induced by environmental fluctuations and improve system stability.

In summary, we have presented a loop-based, time-multiplexed ONN architecture that tackles the key challenges of scalability, reconfigurability, and all-optical operation in optical neuromorphic computing. As all neurons and layers share the same photonic hardware, the scheme avoids inefficient OE conversions in NLAF units, keeps losses constant across layers, and notably simplifies system complexity. Our experiments highlight the ability to realize scalable ONNs, diverse network topologies, and accuracies on par with digital ANNs, while achieving processing latencies several orders of magnitude shorter than a state-of-the-art electronic processor. The ONN can be potentially scaled in both depth and width by leveraging space- or wavelength-domain multiplexing, in combination with enhanced GVD and high-speed modulation techniques. Furthermore, the integration of key components on photonic chips will enable shorter latencies, programmable all-optical NLAFs, and a broader class of network topologies. These results pave the way for general-purpose, large-scale optical neural processors that combine the reconfigurability of electronic platforms with the ultrahigh speed and efficiency of photonics.

## MATERIALS AND METHODS

### Experimental setup

The experimental setup is shown in fig. S12. A continuous-wave (CW) laser source (NKT Laser, Kohearas Basik 1550 nm) is used to generate an optical carrier at 1550 nm. The CW light is first manipulated by an optical switch (10 GHz intensity modulator) driven by a square waveform, where ‘1’ and ‘0’ represent the ‘on’ and ‘off’ states, respectively. When the switch is on, the light passes through the switch for subsequent operations, while the remaining energy is blocked. The input signal (input features) is encoded into the optical domain through the first Mach-Zehnder modulator (MZM1, 40 GHz). Specifically, the input features must be synchronized with the ‘1’ symbol of the switch signal, while the rest of the input signal is set to ‘0’ to further eliminate any leaked CW light from the switch. This ensures that undesired interference between the incoming CW light and the returned optical waveforms is avoided.

The output of MZM1 is connected to one branch of a 50/50 optical splitter, and the other branch is linked with the returned signal (i.e., output of the first layer). The second MZM is used to encode the weight information of the optical neural network (ONN). In the figure shown here, we illustrate the case of a four-layer DNN optimized for the XOR problem, where four groups of weight signals (respectively corresponding to the layers 1 to 4) are separated by a temporal gap equivalent to the delay of the fiber loop (∼400 ns). The resulting temporal products between inputs and weights are then pre-chirped by a 40 GHz phase modulator (PM) driven by a Fresnel time lens (FTL) array, φ(t). Specifically, four groups of Fresnel phase waveforms, each with a different time aperture, are time-multiplexed for implementation of the accumulation operations. Similar to the weight signal, temporal gaps of approximately 400 ns are inserted between these groups. The subsequent group-velocity dispersion (GVD) of ∼12 ns/nm (or ∼ −15,730 ps^2^), implemented using a linearly chirped fiber Bragg grating (LCFBG), aligns the pre-chirped waveforms, coherently accumulating the resulting elementwise products in a serial manner.

Two erbium-doped fiber amplifiers (EDFAs) are used to amplify the optical signals, while an optical tunable filter (OTF) is deployed to improve the signal-to-noise ratio (SNR). Part of the linear output is detected by a photodetector (PD) as the final output of the ONN (linear neurons in the output layer), while the rest is fed into the all-optical nonlinear activation function (NLAF) implemented by a semiconductor optical amplifier (SOA, Thorlabs). The current of the SOA is optimized to realize a nonlinear thresholding operation approximately following a hyperbolic tangent function (Materials and Methods). Another OTF is applied to reduce the out-of-band noise introduced by the SOA. Half the energy of the nonlinear output (e.g., the output of the first, second, or third layer) is detected for the physics-aware training (PAT), while the other half is routed back to MZM2 as the input for the next layer. The switch signal, input waveforms, weight waveforms, and FTL phase waveforms are precisely synchronized using a 1/99 coupler after the PM, with the 1% tap used for synchronization.

### Design of the Fresnel time lens

The Fresnel temporal phase profile φ(t) is constructed by wrapping the target quadratic phase modulation profile of a conventional time lens, $\varphi_{0}t^{2}/2$, within the range [−π,π). This allows us imposing a prescribed high frequency chirp over the required wide temporal aperture (equal to one neuron period, e.g., $t_{r1}$) while avoiding the need for an overall phase modulation excursion that may be otherwise too large for practical implementations, e.g., using electro-optic phase modulation (EOPM). This is a crucial consideration for processing vast time-multiplexed optical data sequences, ultimately allowing for a massive fan-in and highly scalable DNNs (*28*–*30*).

### Calibration of the all-optical nonlinear activation function unit

In the proof-of-concept experiments, we use a commercially available semiconductor optical amplifier (SOA) to provide an all-optical hyperbolic tangent (*tanh*) nonlinear thresholding function. The calibration strategy is shown in Fig. 2A. Five input samples (vectors), each with five input features (or elements), are processed by a 2-layer multilayer perceptron (MLP) consisting of four neurons in the hidden layer and three neurons in the output layer. The input matrix is designed as

$$X=\left[\begin{array}{ccccc} 0 & 0 & 0 & 0 & 0\\ 0.1434 & 0.1091 & 0.25 & 0.1043 & 0.0563\\ 0.2869 & 0.2182 & 0.5 & 0.2086 & 0.1126\\ 0.4303 & 0.3274 & 0.75 & 0.3129 & 0.1688\\ 0.5737 & 0.4365 & 1 & 0.4172 & 0.2251 \end{array}\right]$$ (1)

where each row represents one input sample (five elements), encompassing a total of five samples. The hidden layer comprises four identical neurons, each with the same set of weights

$$W_{h}=\left[\begin{array}{cccc} 0.255 & 0.255 & 0.255 & 0.255\\ 1 & 1 & 1 & 1\\ 0.9367 & 0.9367 & 0.9367 & 0.9367\\ 0.742 & 0.742 & 0.742 & 0.742\\ 0.229 & 0.229 & 0.229 & 0.229 \end{array}\right]$$ (2)

where each column represents the five weights for each neuron. Consequently, the linear output is given by

$$Y_{h,l}=XW_{h}=\left[\begin{array}{cccc} 0 & 0 & 0 & 0\\ 0.4701 & 0.4701 & 0.4701 & 0.4701\\ 0.9403 & 0.9403 & 0.9403 & 0.9403\\ 1.4104 & 1.4104 & 1.4104 & 1.4104\\ 1.8805 & 1.8805 & 1.8805 & 1.8805 \end{array}\right]$$ (3)

where each row represents the output of the corresponding input sample, and the elements in each column increase linearly. Afterwards, the *tanh* function is applied as the nonlinear activation function (NLAF) to rescale the linear outputs, given by $Y_{h}=\text{tanh}\left(Y_{h,l}\right)$.

The weights of the final output layer are designed as

$$W_{o}=\left[\begin{array}{ccc} 1 & 1 & 1\\ 1 & 1 & 1\\ 1 & 1 & 1\\ 1 & 1 & 1 \end{array}\right]$$ (4)

where each column represents the four weights of each neuron. Note that the NLAF is not implemented in the final layer. The SOA can then be calibrated by comparing the normalized experimental outputs with the normalized ideal results.

In the experiment, matrices X, $W_{h}$, and $W_{o}$ are flattened and encoded in the time domain. For instance, the five elements of the first input sample (first row of X) are arranged sequentially along the time domain. The resulting input waveform is then replicated 12 times (4 × 3) times for implementation of the four neurons in the hidden layer and the three neurons in the output layer, while the weight waveform of the hidden layer is replicated 3 times. The design of these waveforms follows the principle described in the main text (Fig. 1). As a result, for one input sample, 12 valid outputs are generated in the hidden layer, and 3 valid outputs are obtained in the final output layer. For the other four input symbols, we can readily time-multiplex all the required waveforms following the aforementioned design rule. As such, the five input samples will result in 60 and 15 valid outputs in the hidden and output layers, respectively, see in Fig. 2 (B and C). Specifically, every 12 outputs in Fig. 2B are in the same voltage level, and the observed variations can be attributed to the noise and other distortions of the waveform generated by the AWG and the radiofrequency (RF) amplifier. Figure 2C shows the output signal from the output layer. Fifteen valid outputs are presented, where every three valid outputs are identical. When the current of the SOA is set to ∼130 mA, the extracted response of the SOA is in an excellent agreement with the response of the ideal *tanh* function, see Fig. 2D. Hence, this is the optimal condition for the SOA to implement the target *tanh* thresholding operation.

### Single-layer perceptron

The implementation of the single-layer perceptron (SLP) is illustrated in fig. S13. The single-layer perceptron (SLP) consists of 200 neurons, each corresponding to one of the 200 classification targets (class 0 for the first neuron, class 1 for the second neuron, and so on). The fan-in size of the perceptron is 200, such that each input vector to be classified consists of 200 randomly arranged binary elements (Fig. 3A). To realize this architecture, the input vector under test must be processed by all 200 neurons, involving a total of 40,000 connections and individual product operations, namely, 200 input elements × 200 neurons. For this purpose, the temporal waveform of each input vector is consecutively replicated 200 times, and the weights of the 200 neurons are encoded in series along the time domain. Specifically, the weights of the first neuron are encoded in the first time slot, synchronized with the first copy of the input vector; the second time-multiplexed neuron is synchronized with the second copy of the input waveform, and so on. The time duration $t_{s1}$ of each input element and weight is ∼22 ps (two sampling points of the AWG), which translates into a total duration of the input vector, and the weight vector corresponding to each neuron, of ∼4.4 ns. However, according to eq. S9 in Supplementary Text, section S1.1, the time aperture $t_{r1}$ of the FTL phase signal (one period) is ∼4.5 ns, determined by the involved $t_{s1}$ and GVD. This is slightly longer than the total duration per neuron. As such, additional null symbols are required to be inserted into each input and weight signal (one neuron), ensuring that their temporal durations match with the temporal aperture of the FTL phase waveform. The same strategy is applied in the multilayer ONN demonstrations reported here. The FTL phase array signal consists of 200 periods, corresponding to the 200 time-multiplexed neurons. Thus, the overall waveform length is ∼900 ns (table S4).

The input vector (blue) and weight waveforms (red) drive the first and second MZMs, respectively, implementing the desired elementwise products. The phases of these temporal waveforms are then manipulated by a PM driven by an FTL phase array signal to implement the subsequent accumulation operations. After the all-optical NLAF unit, the 200 outputs of the SLP are consecutively obtained in the time domain, spaced by a period of ∼4.5 ns. In the example illustrated in fig. S13, the first period shows the highest valid output, indicating that the input vector is recognized as class 0.

### 1D convolutional neural network (CNN)

The convolutional layer performs the convolution between an input vector with 12 features and a kernel of four weights, requiring nine linear operations (fig. S14). To implement the convolution optically, the 12 input features are first segmented into nine time-multiplexed sub-vectors, each comprising four elements over a temporal aperture $t_{r1}$ ∼0.6 ns. These sub-vectors are arranged sequentially in the time domain, with, for example, features 2–5 (blue) following features 1–4 (red). The designed input waveform is then encoded into the optical domain via the first MZM. The kernels are replicated nine times in the time domain, with each copy synchronized to the corresponding sub-vector. This waveform drives the second MZM to encode the weights into the optical domain, implementing the desired elementwise multiplications (the first step in the convolution process). The temporal aperture $t_{r1}$ of each consecutive FTL profile in the phase modulation waveform is ∼0.6 ns. After the FTL-based optical accumulation operation, the desired convolution, including the nine linear operations, is completed, yielding the nine corresponding outputs consecutively along the time domain.

As the FC layer consists of seven neurons, the signals involved in the convolutional layer, including the nine sub-vectors, nine duplicated kernels, and nine FTL phases, are repeated seven times in the time domain, resulting in 7 × 9 = 63 valid outputs after the all-optical NLAF unit for the first layer (fig. S15A). Note that the seven sets of nine outputs are identical. The output waveform of the convolutional (hidden) layer is recirculated as the input to the FC layer, while the weights of the FC layer are shaped in the form of rectangular pulses (pulse width: $t_{s1}$ ∼0.15 ns), each synchronized with the corresponding valid output. A temporal gap of ∼400 ns (the delay of the fiber loop) is inserted to separate the weight waveforms of the first and second layers, ensuring that the returned outputs are aligned with the weight waveforms of the second layer. For example, the nine rectangular weight pulses of neuron 1 in the FC layer are aligned with the nine valid outputs in the first time slot (fig. S14). The resulting time-multiplexed seven linear outputs are detected before the NLAF unit, with the maximum value determining the final prediction. Detailed experimental waveforms are presented in fig. S15.

### Three-layer deep neural network (DNN)

The experimental implementation of the three-layer DNN is shown in fig. S16. Each input vector to be tested is composed of four elements. Consequently, each neuron in the first layer contains four weights and one bias, such that the output of the first layer can be expressed as $f_{\text{NLAF}}\left(x\cdot w+b\right)$, where x is the input vector, w represents the weight vector, and b is the bias. The linear operation can be rewritten as a dot product between the augmented input vector [1,x] and the parameter vector $\left[\begin{array}{c} b\\ w \end{array}\right]$. In this case, to add the bias and the product of x·w optically, the augmented input vector and the parameter vector are required to be encoded into the time domain. Detailed waveforms are shown in fig. S10B, where the element “1” and bias “b” are highlighted by grey boxes. Specifically, the value “1” is prepended to the input vector x along the time axis for implementation of the vector [1,x], while the temporal parameter vector waveform is realized by concatenating the bias and weights in series. The augmented input vector is replicated by 2 × 3 × 3 = 18 times in the time domain as there are two neurons, three neurons, and three neurons in the first, second, and third layers, respectively. Similarly, the parameter waveforms corresponding to the two neurons in the first layer are replicated by 9 times. The subsequent layers only contain weights, such that the design of the corresponding temporal waveforms follow the described general strategy, as illustrated in Fig. 1C (main text). For instance, the weights of the second neuron layer are designed as rectangular pulses, each aligned with the corresponding valid output (fig. S16C). Note that a temporal gap (∼400 ns) is again inserted between the waveforms representing adjacent neuron layers for a proper synchronization among the valid outputs and the corresponding weight waveforms. The time apertures $t_{\mathrm{ri}}$ and temporal resolution $t_{\mathrm{si}}$ implemented for each layer are provided in the caption of fig. S16.

### Four-layer DNN

The experimental implementation of the four-layer DNN is illustrated in fig. S17A. As each layer contains 2 neurons, the input (e.g., [1, 1]) should be replicated 2^4^ = 16 times according to the principle illustrated in Fig. 1C. The input temporal waveform (starting at ∼24 ns) is first synchronized with the weight (yellow) and FTL phase (purple) signals of the first neuron layer. Specifically, the weight and phase modulation waveforms of the two neurons in the first layer are replicated by 8 times for implementation of the following neuron layers (fig. S17B). The output waveform of the first layer (starting at ∼424 ns) is recirculated as the input of the second layer (fig. S17B). Again, a temporal gap of ∼400 ns is introduced to align the returned input waveform with the weights of the second layer, see fig. S17, B and C. The third and fourth layers are implemented following this same strategy (fig. S17, D and E). In the example shown in fig. S17A, the valid output (red dot) in the first period achieves the highest value among all periods, indicating that the prediction of the input vector is class 0 (True). The time aperture $t_{\mathrm{ri}}$ and temporal resolution $t_{\mathrm{si}}$ of each neuron layer are provided in the caption of fig. S17.

### Roundtrip time of the optical fiber loop

To measure the roundtrip time, the waveform propagating through the looped optical system is recorded using a real-time oscilloscope (Agilent, 28 GHz, 80 GS/s). The time interval between the outputs from the first and second roundtrips corresponds to the time delay of the optical fiber loop. This was measured to be ∼400 ns, as shown in figs. S15A to S17A. We estimate that this latency is mainly introduced by (i) pigtails of the benchtop components (∼140 ns), (ii) the LCFBG used for GVD-induced accumulation, which can be shown to introduce a latency equivalent to the temporal aperture $t_{\mathrm{rl}}$ (Supplementary Text, section S6), and (iii) two EDFAs (*52*, *53*) (∼150 ns to 300 ns). As such, the roundtrip time of the current setup could be substantially reduced by (i) optimizing the length of the pigtails and/or (ii) using a high-power CW laser (*54*, *55*), low-loss modulators (*56*), or a high-gain SOA (*57*) for implementation of the NLAF unit, in such a way that the two EDFAs may not be necessary. Based on these strategies, the roundtrip time could be potentially minimized to ∼$t_{\mathrm{rl}}$, namely, the latency introduced by the dispersive line. Importantly, for the multilayer ONNs, the optimized roundtrip time should be longer than the maximum waveform duration per layer (>7 ns for all experiments reported here) to avoid any undesired overlapping between the recirculated signal (output) and the upcoming waveforms (input) that are in the same layer (fig. S9). Note however that this requirement is not necessary for the SLP, such that in this case, the minimum computational latency is given by the waveform duration plus the dispersive line latency. Further discussions on the measured overall latencies and our estimates concerning the system optimized latency performance, including waveform durations, are provided in the Supplementary Text, section S5, with the main results summarized in table S4.

## Supplementary Materials

### The PDF file includes:

Supplementary Text Figs. S1 to S17 Tables S1 to S5 Legend for movie S1 References
