# Supplementary material for: Programmable photonic neural engine with all-optical nonlinear activation and 40,000 connections
Source: Sci Adv. 2026 Aug 19;12(34):eaee9649. doi: 10.1126/sciadv.aee9649 (PMC13488885; doi:10.1126/sciadv.aee9649)
Supplement: Supplementary file 1 [file sciadv.aee9649_sm.pdf]

Science Advances
AAAS

Supplementary Materials for

Programmable photonic neural engine with all-optical nonlinear activation and 40,000 connections

Hao Sun *et al*.

Corresponding author: José Azaña, jose.azana@inrs.ca

Sci. Adv. **12**, eaee9649 (2026)
DOI: 10.1126/sciadv.aee9649

The PDF file includes:

- Supplementary Text
- Figs. S1 to S17
- Tables S1 to S5
- Legend for movie S1
- References

Other Supplementary Material for this manuscript includes the following:

- Movie S1

Supplementary Text

Section S1. Principle of the proposed optical neural network (ONN)

1.1. Physical interpretation and mathematical analysis of the optical MAC operation

The optical multiply-accumulate (MAC) operation involves two mathematical steps: (i) elementwise multiplication (symbol: $\odot$) between inputs and weights; and (ii) coherent accumulation of the resulting products, as shown in Fig. S1. The optical implementation of the multiplication involves two cascaded modulation units, while the accumulation is achieved through a temporal interference mechanism using a Fresnel temporal lens (FTL) and a group velocity dispersion (GVD) medium. To facilitate understanding, we first describe the physical mapping between the logical variables (inputs and weights) and the optical signals. Consider two vectors, **X** (inputs) and **W** (weights) each with m elements, written as:

$$\mathbf{X} = \begin{bmatrix} x_1 \\ \vdots \\ x_m \end{bmatrix},\ \mathbf{W} = \begin{bmatrix} w_1 \\ \vdots \\ w_m \end{bmatrix}. \tag{S1}$$

To perform analog optical computing, these two vectors should be first converted into two temporal optical signals, which is achieved by driving two cascaded electro-optic Mach–Zehnder modulators (MZMs) with the appropriate electronic waveforms. Recall that a MZM enables modulation of an optical signal (a continuous light wave in our case) with a driving electronic waveform, in such a way that the transmission of the MZM is governed by the voltage-induced phase shift between two interferometer arms (*58*). The resulting temporal modulation signals can be mathematically expressed as

$$X(t) = \sum_{k=1}^{k=m} X(k) \cdot rect\left(\frac{t}{t_s} + k\right), W(t) = \sum_{k=1}^{k=m} W(k) \cdot rect\left(\frac{t}{t_s} + k\right), \tag{S2}$$

where t_s is the temporal duration of each of the elements in **X** and **W**. In this implementation, both the input features and the weights are encoded as time-dependent modulation signals. Each element of the vectors is assigned to a distinct temporal slot, t_s, forming a time-multiplexed representation. The ideal modulation process in each MZM device implements a coherent multiplication of the optical electrical field. Specifically, each MZM is biased at V_π, corresponding to a π phase difference between the interferometer arms of the modulator device. Under this condition, the device operates at the zero-crossing point of the optical electric field, where the output field varies approximately linearly with the applied voltage. This latest specification is valid as long as the voltage swing is kept sufficiently small, within the modulator's dynamic range. As a result, the modulated signal can take both positive and negative values, corresponding to opposite optical phases. This enables direct encoding of signed input features and weights in the optical electric field. Importantly, the local slope of the MZM transfer function is negative at V_π, leading to an inversion of the output optical field with respect to the input electrical signal. To ensure that the modulated optical field accurately represents the desired signed input features, this inversion is compensated for by appropriately preconditioning the electrical drive signals. It should be also noted that for the XOR task shown in Fig. 4 of the main text, the inputs (0 and 1) are mapped to normalized driving voltages of −1 and +1 to fully utilize the dynamic range of the modulator and

operate in a high-extinction-ratio regime. In this case, the input MZM is biased at $V_\pi/2$, such that the optical output corresponds to distinct logical levels (i.e., 0 and 1) after modulation.

Following modulation of the signal elements $(x_1, x_2, \dots, x_{m1})$, the weights $w_1, w_2, \dots, w_{m1}$ are encoded by a second, synchronized MZM, also biased at V_π. As the optical signal propagates through the two cascaded modulators, the resulting optical field in each temporal slot becomes proportional to the product of the input feature and the weight, since each modulator scales the field amplitude (including its sign). Therefore, elementwise multiplication is directly realized in the optical field domain through the cascaded modulation process. Mathematically, the envelope of the optical signal after the second modulation unit can be written as $A_e(t) = X(t)W(t)$, and the corresponding optical signal can be expressed as $A(t) = A_e(t)e^{j\omega_c t+\theta}$ where ω_c and θ represent the angular frequency and the initial phase of the continuous-wave light, respectively. For simplicity and without loss of generality, we assume $\theta = 0$. Each encoded feature–weight pair occupies a temporal duration of t_s, and the full product vector is serialized over a total duration $t_r = mt_s$.

Subsequently, a FTL is utilized to pre-chirp the optical signal $A(t)$ with a quadratic temporal phase (i.e., a time-dependent phase shift), $\varphi_0 t^2/2$ (wrapped into $[-\pi, \pi)$). In this expression, φ_0 represents the phase coefficient of the imposed quadratic phase profile, also called the FTL's frequency chirp. In practice, the FTL can be implemented through an electro-optic (EO) phase modulator (PM). The imposed quadratic phase modulation maps different temporal positions to different instantaneous frequencies, generating a linearly chirped optical signal: $B(t) = B_e(t)e^{j\omega_c t}$, where $B_e(t) = A_e(t)e^{j\varphi_0 t^2/2}$, see in Fig. S1. Note that the FTL phase modulation is implemented over the total time duration of the product sequence (or vector) $A_e(t)$, namely, $t_r = mt_s$, such that t_r is usually referred to as the time aperture of the FTL. Through this procedure, the different elements of the vectors X and W are encoded sequentially along the time axis, and they are carried by linearly increasing frequency components, as illustrated in Fig. S1. Subsequently, a suitably designed GVD unit is utilized to synchronize and accumulate the frequency components of the incoming frequency chirped signal $B(t)$, leading to the desired summation of the time-mapped data in the original product vector via temporal interference. This summation is implemented over the temporal aperture t_r. Mathematically, the GVD unit is modelled as a linear spectral phase filtering process, which imposes a frequency-dependent phase shift on the optical field. Specifically, the GVD introduces a quadratic spectral phase of the form $H(\omega - \omega_c) = e^{-j[\ddot{\phi}(\omega-\omega_c)^2/2]}$, where $\ddot{\phi}$ is the dispersion coefficient. Note that in this expression, we have not included the linear phase variation corresponding to the average group-delay introduced by the GVD unit. This quadratic spectral phase corresponds to a frequency-dependent group delay, meaning that different frequency components of the signal propagate at different velocities and therefore experience different time delays. As a result, the frequency components generated by the FTL are temporally realigned after propagation through the GVD element when the dispersion exactly matches the inverse of the lens frequency chirp, i.e., $-1/\ddot{\phi} = \varphi_0$. This phase alignment ensures that the different components interfere constructively at the center of each period (focal point), leading to coherent addition of their amplitudes. Consequently, the optical field at this focal point becomes proportional to the summation of all elements in the product vector along the FTL's temporal aperture, thereby implementing the accumulation operation of the neuron. Given the expression of the transfer function $H(\omega - \omega_c)$, the temporal impulse response of the GVD can be expressed as $h(t) = e^{j(t^2/2\ddot{\phi})}e^{j\omega_c t}$, without considering a constant

proportionality factor of no relevance to our discussions. The temporal waveform after the GVD unit can then be written as:

$$V(t) = B(t) * h(t) = e^{j\omega_c t} \int_0^{t_r} A_e(\tau) e^{j\varphi_0 \tau^2/2} e^{j((t-\tau)^2/2\ddot{\phi})} d\tau, \tag{S3}$$

Where the symbol '$*$' represents a temporal convolution. When $-1/\ddot{\phi} = \varphi_0$, the output signal can be expressed as:

$$V(t) = e^{j(\omega_c t + t^2/2\ddot{\phi})} \int_0^{t_r} A_e(\tau) e^{-jt\tau/\ddot{\phi}} d\tau. \tag{S4}$$

The waveform in Eq. (S4) is temporally centered at t = 0. In practice, the waveform is additionally delayed by the group-delay of the GVD unit at the optical frequency ω_c. At the center $(t = 0)$ of this waveform, the output value is proportional to the integral of $A_e(\tau)$ over the temporal aperture of the FTL:

$$V(0) = \int_0^{t_r} A_e(\tau) d\tau, \tag{S5}$$

The resulting value $V(0)$ (the first red dot) is then proportional to the summation of the elements in the product vector, **X⊙W,** as they are consecutively encoded along the temporal envelope of the optical waveform $A(\tau)$.

Let us consider now the scenario with multiple optical neurons. In this case, the two vectors can be expressed as $\mathbf{X} = [X_1, X_2, \cdots, X_n]^T$ and $\mathbf{W} = [W_1, W_2, \cdots, W_n]^T$, where n represents the nth neuron. The first two sub vectors are presented in Fig. S1 as an example. Recall that the valid output data should be at the center of each period, such that the corresponding temporal locations of the valid data along the time axis are given by:

$$t_\nu = (\nu - 1) m t_s, \tag{S6}$$

where m represents the number of elements in each sub vector, t_s is the temporal duration of each of these elements, and $\nu = 1, 2, \cdots, n$ is a discrete index that indicates the neuron number. Note that we set the temporal location of the first valid output data at zero. To be synchronized with the periodic vector signal, the Fresnel phase profile should be temporally periodic, with a period equal to the time lens aperture, $t_r = m t_s$. Consequently, based on Eq. (S4), the expression of the valid output result for the ν-th period is as follows:

$$V_\nu(t) = B_\nu(t) * h(t) = e^{j(\omega_c t + \frac{(t-t_\nu)^2}{2\ddot{\phi}})} \int_0^{t_r} A_{e,\nu}(\tau) e^{-j(t-t_\nu)\tau/\ddot{\phi}} d\tau. \tag{S7}$$

Note that $B_\nu = A_{e,\nu}(t) e^{j\varphi_0 (t-t_\nu)^2/2} e^{j\omega_c t}$ and $A_{e,\nu}(t) = X_\nu(t) W_\nu(t)$. For the valid output data, located at $t = t_\nu = (\nu - 1) m t_s$, Eq. (S7) can be rewritten as:

$$V_\nu(t_\nu) = e^{j\omega_c (\nu-1) m t_s} \int_0^{t_r} A_{e,\nu}(\tau) d\tau. \tag{S8}$$

<u>1.2. Strategy for designing the ONN and related performance trade-offs</u>

In a practical implementation, the design of the ONN is determined by the amount of the GVD and the sampling rate of the arbitrary waveform generator (AWG) that is used to generate inputs, weights, and phase modulation waveforms. The main design condition derived above, $-1/\ddot{\phi} = \varphi_0$, can be written down in a more convenient manner (*28*), namely,

$$|\ddot{\phi}| = \frac{{t_{ri}}^2}{m_i 2\pi} = \frac{t_{ri} t_{si}}{2\pi} = \frac{m_i\, {t_{si}}^2}{2\pi}, \tag{S9}$$

where i denotes the ith layer, and m_i is an integer. In general, this condition must be satisfied for all layers in the network. Eq. (S9) can be easily obtained by noting that the magnitude of the chirp factor φ_0 of the time lens modulation is given by the ratio of the modulation's radial frequency bandwidth ($\Delta\omega_i$) to its time aperture (t_{ri}). In turn, $\Delta\omega_i = \frac{2\pi}{t_{si}}$, where t_{si} is an estimate of the minimum temporal feature, or time resolution, of the phase modulation function, e.g., as limited by the AWG used for generation of this modulation function.

According to Eq. (S9), for the first neuron layer, a larger fan-in size m_1 can be achieved by use of a higher GVD or a shorter modulation resolution (faster AWG). As described above, the FTL introduces a periodic frequency chirp with a bandwidth of $\Delta\omega_1 = |\varphi_0| t_{r1}$ across the incoming optical waveform. After propagating through the GVD unit, the pre-chirped waveform is compressed to a transform-limited waveform, extending over a frequency bandwidth $\Delta\omega = \Delta\omega_1$ and centered at the temporal focal point (valid output), see Fig. S1. Consequently, the temporal resolution δt, or shortest time feature, of the valid output is approximately given by $2\pi/\Delta\omega_1 = t_{s1}$ (resolution of the time-lens modulation). This indicates that the pulse width of each of the weight waveforms in the second layer (for the multilayer scenario) should be fixed to $\sim t_{s1}$, as this ensures that the valid output is captured over the longest possible duration, hence maximizing energy efficiency, while discarding all other undesired information along this output waveform.

For the following neuron layers, the temporal apertures t_{ri} of the ith layer are directly determined by $t_{ri} = \prod_{k=1}^{k=i-1} n_k t_{r1}$, where n_k is the number of neurons in the kth layer. When scaling up the ONN in depth (more layers) and width (more neurons per layer), the temporal aperture t_{ri} of the time-lens waveform is increasingly broadened, requiring a wider time-lens bandwidth $\Delta\omega_i$ and a corresponding finer temporal resolution t_{si}. In practice, the shortest t_{si} is limited by the operation bandwidth or sampling rate of the AWG, which, in turn, determines the longest temporal aperture that can be handled within the neural network, and thus the maximum depth and width of the ONN. On the other hand, a wider bandwidth of the time-lens phase modulation waveform will translate into a narrower temporal resolution $\delta t = t_{si}$ of the valid output, such that the pulse width of the weight waveforms must be adjusted accordingly for each neuron layer.

1.3. Temporal aperture and fan-in capacity

The temporal aperture t_r plays a critical role in determining the accuracy and scalability of the optical MAC operation. Since each element occupies a temporal slot of duration t_s, the total duration of the time-multiplexed signal is $t_r = m t_s$, where m is the number of elements (i.e., the fan-in).

The product signal $A_e(\tau)$ has an approximate bandwidth $\Delta\omega \sim 2\pi/t_s$. Through the frequency-to-time mapping implemented by the FTL process ($t = \omega\ddot{\phi}$), this bandwidth is mapped into a temporal spread of approximately $\Delta t = \Delta\omega|\ddot{\phi}| \sim 2\pi|\ddot{\phi}|/t_s$ at the output. To ensure accurate

accumulation, the temporal aperture must be sufficiently large to accommodate this mapped signal, namely, $t_r \geq \Delta\omega|\ddot{\phi}|$. If the temporal aperture is smaller than this value, high-frequency components will extend beyond the available time window and overlap with adjacent periods, leading to distortion and degradation of the MAC accuracy. Here, we select the minimum allowable value $t_r = \Delta\omega|\ddot{\phi}|$ to minimize the operation latency while ensuring that no temporal overlap occurs.

Hence, the temporal aperture is directly related to the fan-in capacity of the system, according to the Eq. (S9). For a given group-velocity dispersion ($\ddot{\phi}$), the number of elements (fan-in) m that can be processed is determined by how many temporal slots of duration t_s can fit within this window, i.e., $t_r = mt_s$. In practice, the minimum achievable t_s is limited by the sampling rate of the arbitrary waveform generator (AWG). Therefore, when the group-velocity is determined, a faster AWG sets the shorter slot duration t_s, which in turn contributes to a larger fan-in m.

Section S2. Physics-aware Training

2.1. Principle of the physics-aware training

A highly efficient physics-aware training (PAT) method is applied to train the proposed ONNs(*9*, *24*). The training of the three-layer DNN (Fig. 4A in main text) is illustrated in Fig. S2 as an example. The output signals of the hidden layers and the final output layer are detected after the all-optical NLAF unit and the GVD unit, respectively, as shown in Fig. S2a. According to the DNN topology shown in Fig. 3a (main text), the final output waveform contains three valid outputs ($x^{[4]}$), respectively corresponding to the likelihood of the three classes in the Iris dataset (Fig. S2b bottom). In terms of the first hidden layer, the two outputs are replicated nine times along the time domain, resulting in 18 ($2 \times 3 \times 3$) valid outputs ($x^{[2]}$), while the three outputs of the second hidden layer are replicated three times, generating 9 (3×3) valid outputs ($x^{[3]}$) (Fig. S2b).

The PAT algorithm is illustrated in Fig. S2c. Specifically, the three blue boxes represent the feedforward propagation of the data stream in the optical neuron layers, generating three groups of outputs ($x^{[2]}, x^{[3]}, x^{[4]}$) applied directly in the training. First, the three final outputs ($x^{[4]} = [p_1, p_2, p_3]$) are compared with the targets ($[y_1, y_2, y_3]$) to calculate the cross-entropy loss (L)(*59*). The backpropagation of the PAT algorithm follows the same chain rule as the digital ANNs, but $x^{[2]}$, $x^{[3]}$, and $x^{[4]}$ are taken directly from the experimental measurements. Differentiable functions are then used to emulate the physical linear and nonlinear operations, allowing the physical system to be trained end-to-end. Specifically, the differentiable functions of each layer are expressed as:

$$\begin{aligned} x^{[2]} &= f_1\left(x^{[1]}, w^{[1]}, b^{[1]}\right) = tanh\left(x^{[1]} \cdot w^{[1]} + b^{[1]}\right), \\ x^{[3]} &= f_2\left(x^{[2]}, w^{[2]}\right) = tanh\left(x^{[2]} \cdot w^{[2]}\right), \\ x^{[4]} &= f_3\left(x^{[3]}, w^{[3]}\right) = x^{[3]} \cdot w^{[3]}, \end{aligned} \tag{S14}$$

where "·" denotes the dot product (multiple MAC operations) between vectors $x^{[i]}$ and $w^{[i]}$, and "$tanh$" is the hyperbolic tangent function representing the nonlinear response of the SOA.

The central task in training is to compute gradients ($g_{w^{i=2,3}}$ or g_{w^1,b^1}) with respect to the weights and biases. Backpropagation begins at the output layer. The gradient of the output layer is computed as

$$g_{w^{[3]}} = \frac{L}{\partial w^{[3]}} = \frac{L}{\partial x^{[4]}}\frac{\partial x^{[4]}}{\partial w^{[3]}}, \tag{S15}$$

where $g_{x^{[4]}} = \frac{L}{\partial x^{[4]}}$. The weights in the output layer are then updated as $w^{[3]} \rightarrow w^{[3]} + \alpha g_{w^{[3]}}$, where α is the learning rate. We follow the same procedure to train the second and the first layer, considering that the gradients of $g_{w^{[2]}}$, $g_{w^{[1]}}$, and $g_{b^{[1]}}$ can also be obtained using the chain rule, as follows:

$$g_{w^{[2]}} = \frac{L}{\partial w^{[2]}} = \frac{L}{\partial x^{[4]}}\frac{\partial x^{[4]}}{\partial x^{[3]}}\frac{\partial x^{[3]}}{\partial w^{[2]}}$$
$$g_{w^{[1]}} = \frac{L}{\partial w^{[1]}} = \frac{L}{\partial x^{[4]}}\frac{\partial x^{[4]}}{\partial x^{[3]}}\frac{\partial x^{[3]}}{\partial x^{[2]}}\frac{\partial x^{[2]}}{\partial w^{[1]}}$$
$$g_{b^{[1]}} = \frac{L}{\partial b^{[1]}} = \frac{L}{\partial x^{[4]}}\frac{\partial x^{[4]}}{\partial x^{[3]}}\frac{\partial x^{[3]}}{\partial x^{[2]}}\frac{\partial x^{[2]}}{\partial b^{[1]}} \tag{S16}$$

2.2. Hyperparameters tuning

In our experiments, the PAT is implemented through the PyTorch package using the cross-entropy loss (*59*) and Adam optimizer (*60*). The initial learning rate was set to 0.01 and adjusted using ReduceLROnPlateau function (*61*) (factor = 0.5, patience = 1), such that the learning rate is reduced by half when the validation loss is not improved. A relatively higher learning rate is implemented to accelerate the training process. The training is terminated if the validation accuracy remains unchanged for three training epochs. This is known as the early-stopping training strategy to prevent overfitting (*62*). The batch size is fixed at 1. The dataset splitting is summarized in Table S1.

To further assess the robustness and generality of the proposed training pipeline, we conducted additional experiments using a larger batch size. Specifically, we implemented a two-layer MLP (3 neurons per layer) on the Iris dataset with a batch size of 10. In this configuration, 10 input samples and their corresponding weight signals are sequentially encoded and processed along the time domain within a single training iteration, as illustrated in Fig. S3. The training dataset contains 90 samples, and validation/test dataset each involves 30 samples. The corresponding training results are shown in Fig. S4. Across three independent runs with different random initializations, the model exhibits consistent convergence behavior. In the first two runs, the validation accuracy reaches 100% at epoch 23 and 17 respectively, and with corresponding test accuracies of 100%. In the third run, the validation accuracy converges to 90% at epoch 28, with a matching test accuracy of 90%. In all cases, training is terminated once convergence is achieved (early stopping). For comparison, the corresponding digital model is trained 50 times over 30 epochs. The experimental results of the ONN are comparable to those obtained from the digital counterpart, further supporting the robustness of the proposed training approach.

Section S3. Results of digital neural networks

To benchmark performance, the four ANN topologies were also implemented digitally on an electronic processor. Training was carried out in PyTorch with datasets organized as for the corresponding optical implementations, as detailed in Table S1. The CrossEntropyLoss function and the Adam optimizer were used, with the learning rate adaptively adjusted via the

ReduceLROnPlateau function based on the validation loss. Each model was trained 30 times to ensure robustness and reduce the influence of random fluctuations on test accuracy (*63*). Additional hyperparameters, including the initial learning rate and number of training epochs, are listed in Table S2. The training, validation, and testing results are shown in Fig. S8.

Section S4. Estimation of the signal-to-noise ratio and effective number of bits

The computation precision of the experimental setup is quantified using the effective number of bits (ENOB), which is a standard metric for evaluating precision in analog and mixed-signal systems. The ENOB is related to the signal-to-noise ratio (SNR) and given by (SNR – 1.76)/6.02 (*39*). To evaluate the effective precision of our optical neural network (ONN) implementation, we extract the SNR directly from experimentally measured output waveforms. As a representative example, we analyze the four-layer ONN output. The temporal output waveform of the fourth layer is recorded using an 80 GS/s real-time oscilloscope (Fig. S6b, top panel). To accurately estimate the SNR, we separate the measured waveform into two components: (i) the signal-dominant region and (ii) the noise-dominant region, as illustrated in Fig. S6c (top) and Fig. S6a (top), respectively. This segmentation allows us to independently estimate the signal and noise contributions from the same experimental dataset, from which we obtain the SNR of the measured waveform.

The total measured power, which includes both signal and noise contributions, is obtained from the power spectral density (PSD) of the full waveform (Fig. S6c), yielding $P_{total}= P_{sig}+ P_{noise}$. Since direct extraction of in-band noise from the signal-containing waveform is challenging, we estimate the noise power (P_{noise}) from the dedicated noise-only segment shown in Fig. S6a. This segment captures the cumulative noise contributions from all experimental sources, including detector, electronic/optical amplifier, and system fluctuations under identical acquisition conditions. From this analysis, we obtain SNR= $P_{sig}/P_{noise}=(P_{total}- P_{noise})/P_{noise}$ ~ 29.31 dB, where P_{total}*=2.1602 (a.u.),* P_{sig}*=2.1577 (a.u.), and* P_{noise}*=0.0025 (a.u.).* Substituting this value into the ENOB expression yields: ENOB of ~ 4.58 bits. This level of precision is sufficient for neural network inference and training tasks in analog photonic computing systems, where typically more than 4-bit effective precision is considered adequate for reliable machine learning performance (*26*). As a comparison, the arbitrary waveform generator (AWG) used in the experiment has an ENOB of approximately 5.4 bits (*64*).

Section S5. Estimation of the latency

A detailed estimation of the latency is summarized in Table S3. Specifically, the overall latency of the ONN includes (i) the latency of the fiber loop, given by lT, where T is the loop roundtrip time, and l is the total number of layers or roundtrips through the loop (Fig. S7), plus (ii) the temporal duration Δt of the waveform per layer. As the temporal duration is identical for each neuron layer, we simplify the notation by presenting the expression only for the output layer, $\Delta t = n_l t_{rl}$, where n_l is the number of neurons in the output layer, and t_{rl} is the temporal aperture in the output layer given by $t_{rl} = \prod_{i=1}^{i=l-1} n_i t_{r1}$ (defined in Section 7).

The loop roundtrip time could be notably reduced using the strategies described in the main article and Methods. Specifically, the minimum physical roundtrip time is determined by the latency introduced by the LCFBG in the fiber loop. This latency can be expressed as $\Delta\omega_l \cdot |\ddot{\phi}|$, where $\Delta\omega_l$ is the bandwidth of the temporal waveform in the output layer (broader bandwidth waveform across all the network). Considering that $\Delta\omega_l = |\varphi_0| t_{rl}$ and $|\varphi_0| = 1/|\ddot{\phi}|$, as shown in Supplementary Section 1.2, the latency of the LCFBG is then equal to the temporal aperture t_{rl}. As such, the LCFBG provides a substantially shorter latency than that of a single mode fiber

introducing the same amount of GVD. To achieve the optimal latency in the network, the fiber loop length should just be adjusted to match the output waveform duration per layer $n_l t_{rl}$, such that the overall latency is then given by $(l+1)n_l t_{rl}$. As mentioned in the Methods, this general estimate is only applicable to multilayer ONNs. For the single-layer perceptron (SLP) case, the overall latency can be minimized to around $n_l t_{rl}$ (waveform duration) + t_{rl} (the minimal physical latency introduced by the GVD line). The corresponding optimized overall latencies are listed in Table S3.

The temporal waveform durations ($n_l t_{rl}$) of the ONN topologies demonstrated in our experiments are listed in Table S3. These durations can be further reduced by using a smaller amount GVD and an AWG with a higher sampling rate. For instance, with a 256 GS/s AWG and a 4-mm-long waveguide Bragg grating (GVD ~510 ps/nm) (*40*), the maximum temporal aperture could be shortened to ~1.1 ns. Under these conditions, the estimated waveform duration per layer would be approximately an order of magnitude shorter than those in the current setup. However, as discussed above, the use of a reduced GVD would constrain the scalability of the ONN. For instance, a SLP with a large fan-in (200) could not be implemented using the considered reduced GVD.

For comparison, digital processing latencies were estimated using an NVIDIA A100 GPU (*65*). Only linear and nonlinear operations were considered, excluding initialization and system-level overheads. A warm-up phase was performed prior to measurement to eliminate transient effects such as CUDA kernel cold starts and memory allocation. Latency was then averaged over 10,000 repeated inferences per test sample to obtain steady-state performance. The average latency per sample is reported in Table S3.

Section S6. Path to full photonic integration

An interesting prospect of the proposed architecture is its potential for full on-chip integration, which would considerably reduce latency and device footprint. The most promising path toward this goal is through silicon photonics (SiP), a mature, CMOS-compatible platform that provides a robust set of all required components in integrated formats, including high-speed modulators (*66*), amplifiers (*43*), photodetectors (*44*), and even all-optical nonlinear units (*47*).

The primary challenge for full integration, however, lies in the implementation of on-chip dispersive lines with a sufficiently large GVD. Current integrated dispersive lines can achieve GVDs equivalent to tens of kilometers of single-mode fiber (*40*, *42*). Consistently with the estimates provided in Section 5, this level of performance is sufficient for integrating the CNN and DNN architectures demonstrated in our work, as described in the main text, confirming the immediate viability of on-chip implementation for deep learning models.

Nonetheless, achieving the massive scalability of our large-scale SLP experiment requires a notably higher GVD. The performance of current SiP-based dispersive lines is fundamentally constrained by propagation loss, which limits the total achievable dispersion. To overcome this bottleneck, future work could leverage emerging low-loss platforms. Materials such as thin-film lithium niobate (TFLN) and silicon nitride (SiN) are highly promising alternatives (*67*, *68*), as their lower intrinsic losses could enable integrated dispersive elements with the high GVD necessary for future large-scale optical neuromorphic processors.

Section S7. Estimation of the scalability

7.1. Single layer perceptron

According to the Nyquist–Shannon theorem, each bit should contain at least two sampling points to ensure that the desired vector waveform is generated from the arbitrary waveform generator (AWG). Assuming a state-of-the-art 256 GS/s AWG (*46*), the shortest time feature t_s that could be generated with this instrument is around 8 ps. Assuming also that the dispersive unit is implemented using a commercially available linearly chirped fiber Bragg grating (LCFBG) providing a GVD of ~250 ns/nm (*45*), a fan-in ~33,305 could be achieved. This estimate is based on Eq. (S9).

7.2. Multilayer neural network

The temporal aperture t_{r1} of the first layer is given by the number of input features (m_1) and the GVD ($\ddot{\phi}$) according to Eq. (S9). Then, we can readily calculate the required temporal resolution t_{s1} of the time-lens phase modulation waveform. Afterwards, the temporal aperture of the output layer is given by $t_{rl} = \prod_{i=1}^{i=l-1} n_i t_{r1}$, where l represents the total number of layers and n_i is the number of neurons in the ith layer. We recall that this specification determines the maximum needed temporal aperture for the time lenses to be implemented in the network. For a given amount of GVD, the time-lens frequency chirp is fixed ($\varphi_0 = -1/\ddot{\phi}$), such that a time lens modulation sweeps a higher frequency bandwidth as it extends over a longer temporal aperture. A higher modulation bandwidth requires a finer temporal resolution (shorter t_{si}), and this is ultimately constrained by the speed or bandwidth of the AWG used to produce the time-lens modulation waveform. In turn, this requirement constrains the scalability of the designed ANN topology, as detailed above in Section 1.2. As the design of the ONN is task oriented, Table S4 provides a rough estimate of the scalability of the ONNs designed for the three reported tasks. All these estimates are based on the aforementioned LCFBG (*45*), providing a GVD of ~250 ns/nm, and a 256 GS/s AWG (*46*). The longest time-lens aperture that could be achieved with these specifications is ~520 ns. Considering this constraint, the ONN could be extended up to eight layers (deeper) for the XOR problem as less neurons are required per layer (narrower). In contrast, the one-dimensional (1D) convolutional neural network (CNN) designed for the vowel recognition task could involve up to 20 neurons (wider) in the second hidden layer, while the network could only be scaled up to three layers (shallower).

7.3. Scalability Enhancement via Hybrid Time– and Spatial-Domain Multiplexing

To address the scalability limitation associated with temporal repetition, we envision the combination of our newly proposed concept based on time-domain photonic processing with other multiplexing strategies (such as spatial or wavelength division multiplexing). As an example, we introduce and describe here an extended architecture that combines time-domain multiplexing with spatial-domain fan-out, as illustrated in Fig. S8. In this hybrid scheme, multiple optical MAC units operate in parallel across N spatial channels, such that several neuron computations can be performed simultaneously. Consequently, the required number of temporal repetitions is reduced by a factor of N, providing a direct pathway to improving scalability while preserving the benefits of time-domain processing.

Figure S8a shows a conventional multilayer perceptron, where each layer consists of multiple neurons fully connected to the next layer. Fig. S8b shows the physical implementation of the proposed hybrid architecture. The input data are first encoded into a temporal optical waveform and then split into N parallel channels using a 1×N splitter. Each channel contains an optical time-processing MAC unit, which performs the weighted multiplication and accumulation through temporal phase modulation followed by the corresponding spectral (dispersive) phase modulation. An additional MZM is incorporated in each channel to select the valid output while suppressing the unnecessary information. The selected outputs are then passed through an optical delay-line

(ODL) array, where channel-dependent delays are applied to align the signals sequentially in time. After recombination using an N×1 combiner, the outputs from all channels form a continuous time-domain sequence, which is subsequently fed into a single NLAF unit to generate the nonlinear neuron outputs.

Figure S8c illustrates the detailed signal flow using a simplified example of a two-layer network with 4 neurons per layer. The input data are encoded into a temporal waveform following the same procedure described in the manuscript, with a temporal aperture t_{r1}. Here we only illustrate two spatial channels (N = 2), meaning that two MAC operations can be executed in parallel. As a result, the input waveform only needs to be replicated twice, rather than four times as required in a purely time-multiplexed implementation. This reduction is highlighted by the portion enclosed in the red dotted box in Fig. S8c. Within the first layer, each channel produces partial MAC outputs that are distributed in time. After valid time-slot selection and channel-dependent delay alignment, the outputs from the two channels are recombined and passed through the NLAF. This process yields 4 nonlinear neuron outputs, denoted as ⑪, ⑬, ⑫, and ⑭, which appear consecutively along the time domain. These outputs are inherently time-multiplexed and serve directly as the input to the second layer.

For the second layer, the temporal aperture per neuron becomes $t_{r2} = 2t_{r1}$. The same optical hardware is reused, with appropriately reconfigured temporal phase modulation to perform the required MAC operations, consistent with the method described in the main text. Following the same sequence of operations—parallel MAC, valid output selection, delay alignment, recombination, and nonlinear activation—the outputs of the second layer (e.g., ㉑, ㉓) are again generated sequentially in time. In order to have 4 neurons output (㉑, ㉓, ㉒, ㉔) in the second layer, the time-multiplexed input sequence from the first layer (indicated by the red dotted box) must be replicated. However, due to the presence of N spatial channels, the number of required repetitions is reduced by a factor of N for each layer. This effectively shortens the total waveform duration and alleviates the scalability constraint associated with pure time multiplexing. Importantly, this hybrid time–space architecture retains the key advantages of the original time-multiplexed approach. In particular, the network topology remains fully reconfigurable through waveform programming, and only a single NLAF unit is required to provide the nonlinearity for all neurons. As such, the proposed scheme offers a balanced trade-off between latency, hardware complexity, and insertion loss, and provides a practical pathway toward scalable and programmable end-to-end optical neural networks.

To validate the capability of the proposed architecture, we perform a numerical demonstration using a 3-layer optical convolutional neural network (OCNN) on the MNIST handwritten digit dataset. The system is designed based on currently available components in our laboratory, including a linearly chirped fiber Bragg grating providing a group-velocity dispersion equivalent to approximately 715 km of standard single-mode fiber, and an arbitrary waveform generator with a sampling rate of up to 120 GS/s. We assume 16 parallel physical channels (N=16), consistent with feasible implementations.

The OCNN architecture is shown in Fig. S9. The input image is first down sampled to 9×9 pixels. A convolutional layer with a 2×2 kernel is applied, producing 64 nonlinear outputs. These outputs are then fed into a fully connected layer with 32 nonlinear neurons, followed by a final output layer consisting of 10 linear neurons (with 10 active channels). The fully connected layers (FC1 and FC2) follow the same operational principle as described in Fig. S8c.

Note that to perform the convolution operation — where the kernel slides across different sub-regions of the image — we adopt an encoding strategy tailored to the hybrid time–space architecture. Specifically, the weight information (i.e., the flattened convolutional kernel) is encoded onto the optical waveform using the first MZM prior to the 1×N splitter, such that an identical weight profile is broadcast to all spatial channels. In contrast, the input image is flattened and serialized in the time domain, and different sub-matrices (corresponding to different local regions of the image used in the convolution) are distributed across spatial channels. In this manner, each channel processes a distinct input sub-region while sharing the same kernel, enabling parallel convolution operations without redundant weight encoding.

With 16 spatial channels operating in parallel, the overall waveform duration is reduced to approximately $\frac{64}{16} \times \frac{32}{16} \times t_{r1} \sim 4.8$ ns, leading to an estimated total latency of ~14.4 ns for the three-layer network. The temporal aperture t_{r1} is determined using Eq. (S9), $|\ddot{\phi}| = \frac{{t_{ri}}^2}{m_i 2\pi}, i = 1$, with $m_1 = 4$ corresponding to the kernel size.

During training, the batch size is set to 64 and the learning rate to 0.01. The dataset is divided into 54,000 training samples, 6,000 validation samples, and 10,000 test samples. To ensure statistical reliability, the training process is repeated 10 times. The simulation results in Fig. S10 show that the proposed ONN achieves an average test accuracy of approximately 95.2%, which is close to the digital baseline of 96.7%. The slight performance degradation is attributed to accumulated errors associated with analog optical computation across multiple layers (*69*), which remains an open topic for further investigation.

Overall, these results demonstrate that, by combining time-domain processing with spatial multiplexing, the proposed architecture can achieve high computational efficiency, low latency, and performance approaching that of digital implementations. This provides a potentially viable pathway toward scalable and programmable end-to-end optical neural networks.

Section S8. Estimation of power and energy consumption

The current implementation of the loop-based ONN relies on commercially available standalone benchtop instruments, which are not optimized for energy efficiency. For example, the 4-channel Keysight AWG consumes approximately 50 W at 92 GS/s, and the real-time oscilloscope (Agilent 28 GHz, 80 GS/s) consumes ~200 W per channel. These values reflect laboratory-grade instrumentation rather than ONN hardware.

To provide a more meaningful comparison with reported end-to-end ONNs, we further estimate the power consumption assuming a chip-scale implementation using compact and integrated photonic/electronic components. Under this assumption, the overall estimated power consumption is approximately ~6 W. The breakdown is as follows:

- *CW Laser*: A 20 mW tunable telecom laser with 20% wall-plug efficiency corresponds to ~0.1 W (*19*).
- *Three 160 GS/s DACs:* Two for MZMs, one for PM, 3×0.251 W (*70*).
- *One 14 GS/s DAC:* For optical switch (MZM), 0.05 W (*71*).
- *Three MZMs with RF drivers (one for optical switch):* 3×0.078W (*72*).
- *Phase modulator (PM):* with $V_\pi \sim 3.1$ V at 1 GHz, and 50 ohms terminator (*56*). To implement the Fresnel time lens (FTL), the peak-to-peak voltage V_{pp} driving the phase

modulator should be over 6.2 V, resulting in a power consumption of ~ 0.105 W according to $P = (\frac{V_{pp}}{2\sqrt{2}})^2/R$ (*41*).

- *RF driver (60 GHz): with output V_{pp} of ~ 6.5 V:* 0.67W (*73*).
- *Two EDFAs: commercially available compact units:* 2×1.5 W (*74*).
- *One SOA (nonlinear activation):* 0.576 W (*75*).
- *Optical receiver (44 GHz):* PD and TIA, 0.216 W (*76*).
- *ADC:* 0.2376 W (*77*).

In our loop-based ONN architecture, repeating the input waveform or modifying the network topology does not increase the power consumption, since the physical hardware remains unchanged. The estimated power consumption of our system is therefore determined primarily by the employed optoelectronic components and is comparable to the best-case estimation in the reported end-to-end ONN systems (~ 5 W) (*19*), as summarized in the Table S5. However, the total energy consumption increases with the operation time, since time-domain multiplexing requires repeated waveform propagation and hence a longer effective processing duration.

To evaluate this effect, we consider a single-layer perceptron (SLP) with 200 neurons, where the input waveform is time-multiplexed and repeated 200 times. This leads to an overall processing latency of 1300 ns in our experiment (considering 400 ns fiber-loop delay). Based on this latency, the corresponding estimated energy consumption is ~7.8 μJ. For comparison, this energy consumption is approximately three orders of magnitude lower than that of an equivalent implementation on an NVIDIA A100 GPU (~3270 μJ), where the estimate is based on a processing latency of 52.7 μs and a static power consumption of ~62 W (Fig. S11). This highlights the potential energy efficiency advantage of photonic time-domain processing for such operations.

Moreover, we also note that scaling to 200 neurons remains challenging in current end-to-end ONNs due to accumulated optical loss. For instance, considering a representative insertion loss of ~0.22 dB per Mach–Zehnder interferometer (MZI) (*19*), implementing the MAC operations for 200 neurons (as in the single-layer perceptron demonstrated in our work) would require 200 MZIs, resulting in an accumulated insertion loss of approximately 44 dB. This estimation does not include additional losses introduced by the corresponding optical nonlinear activation function (NLAF) units, which would further exacerbate the problem. Such a high loss level poses a substantial challenge for practical large-scale implementations.

For completeness, we also compare our system with a representative 3-layer end-to-end ONN reporting ultra-low latency (~ 410 ps) and best-case power consumption (~5 W) (*19*). For our 3-layer system, the estimated worst-case energy consumption is ~7.2 μJ, considering a 400 ns fiber-loop delay, while the best-case energy consumption is ~ 0.3 μJ under an optimized latency configuration. This corresponds to an energy consumption that is still ~150 times higher than that reported in (*19*). We reiterate that this mainly due to the time-domain repetition of the input waveform inherent in our architecture.

Nevertheless, despite this increase in energy consumption relative to fully parallel ONN implementations (for networks involving a relatively modest number of neurons), our current system still offers a notable advantage over digital processors. Even in the worst-case scenario (considering 400 ns fiber-loop delay), the estimated energy consumption remains approximately three orders of magnitude lower than that of an equivalent NVIDIA A100 GPU implementation (~7000 μJ, considering static power consumption only).

Overall, while the proposed time-multiplexed loop architecture introduces higher energy consumption compared with fully parallel optical systems, the time-multiplexed scheme can be scaled up more easily to a larger number of neurons while providing substantial benefits in terms of programmability and reconfigurability. These features are critical for scalable neuromorphic photonic computing but remain challenging to achieve in existing end-to-end ONN architectures. A detailed comparison of power consumption, latency, and system characteristics between our work, state-of-the-art ONNs, and digital processors such as the NVIDIA A100 GPU is provided in the Table S5.

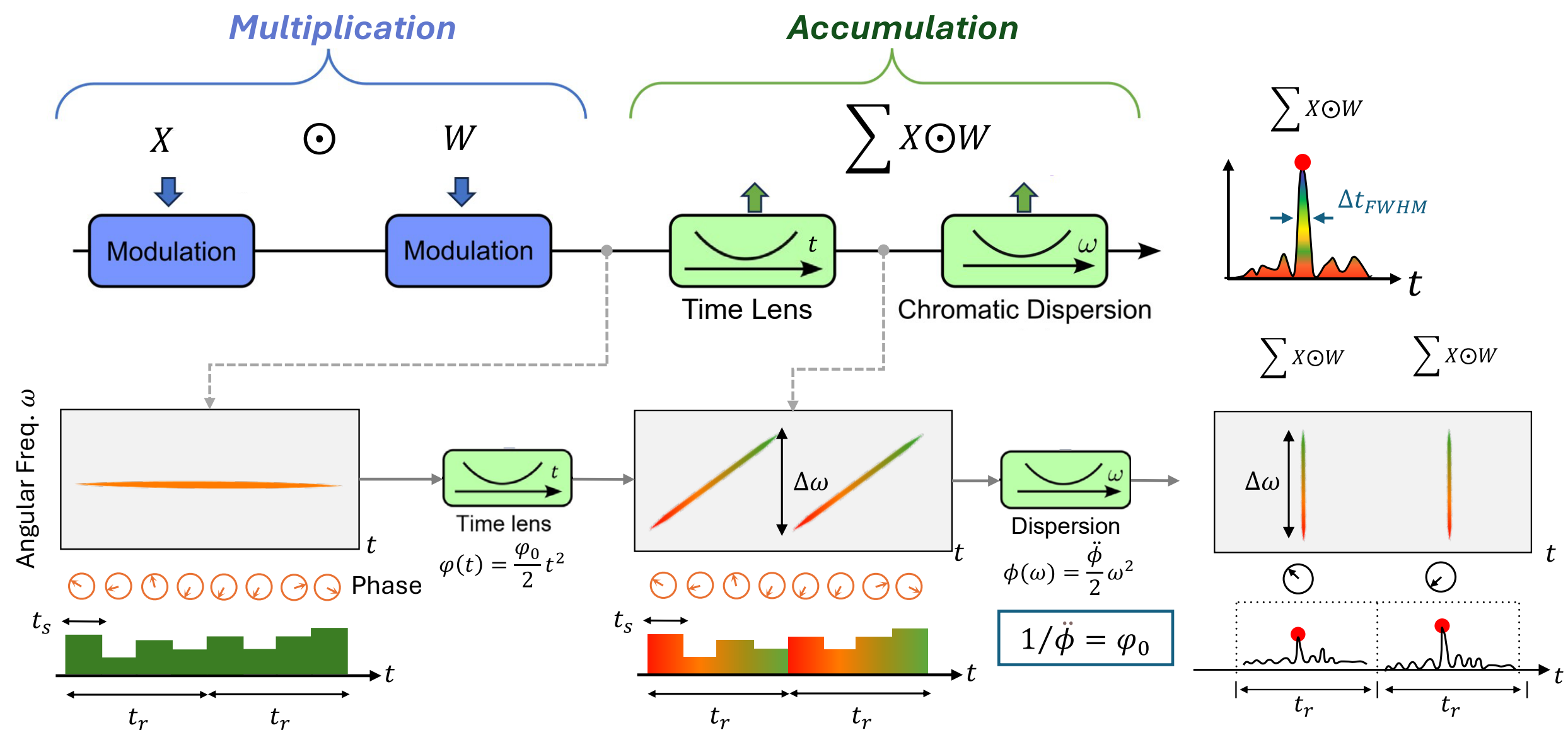

Fig. S1.

Detailed illustration of the optical MAC operation. ⊙ represents the elementwise multiplication. The red dots represent the valid outputs. FWHM: full width at half maximum.

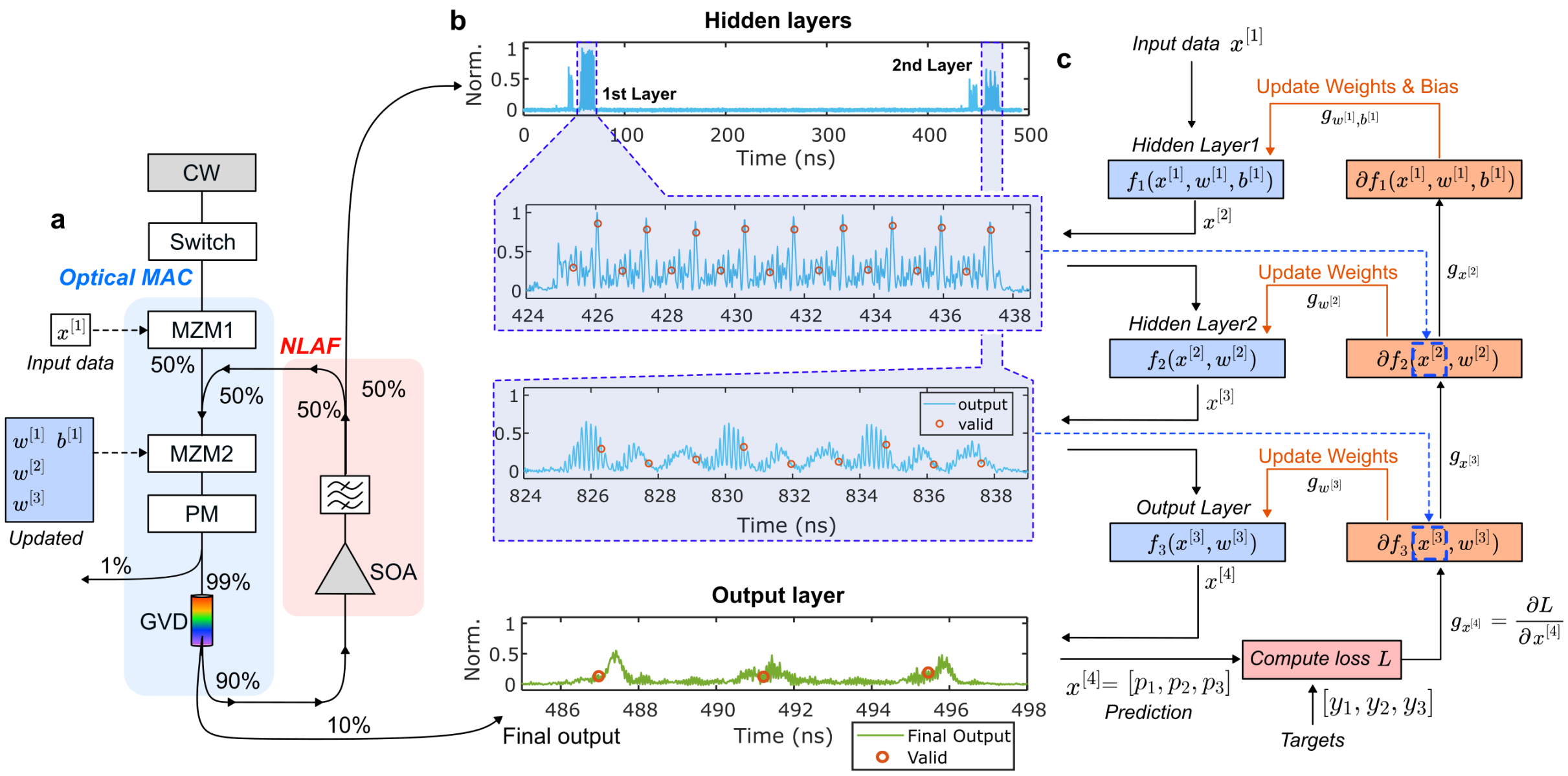

Fig. S2

Illustration of the physics-aware training algorithm. **a,** Experimental setup of the proposed CONN. **b,** Experimental output waveform of the hidden and final output layers. **c,** PAT algorithm for the on-line training.

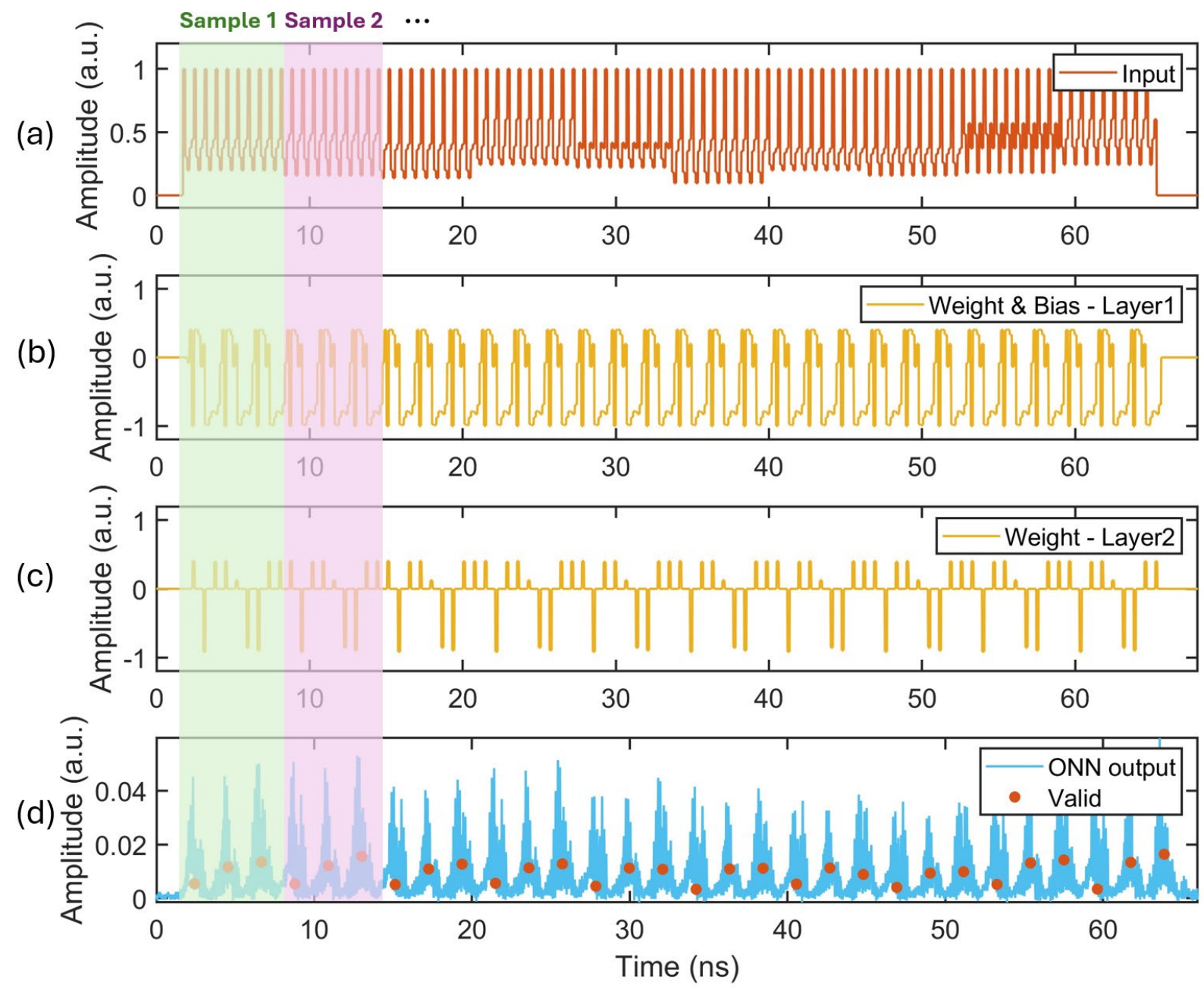

Fig. S3.
Measured waveforms of the 2-layer multilayer perceptron. a-c, The designed input and weight signals. The first layer contains the bias. **d**, the output of the ONN.

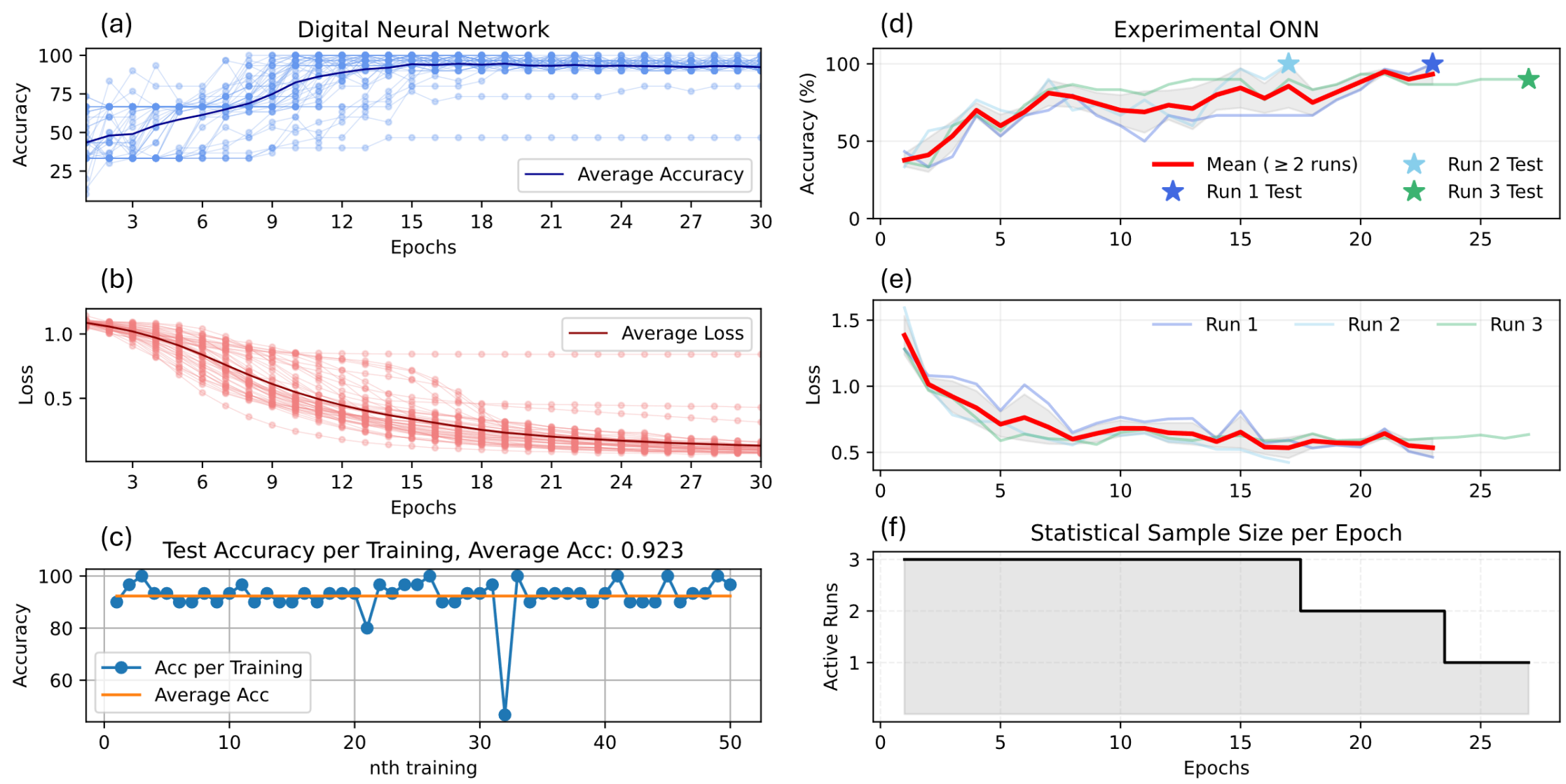

Fig. S4.

The training results of the digital neural network and the ONN. a-c, The validation accuracy, training loss, and the test accuracy of the digital neural network. **d-f**, The validation accuracy, training loss, and active number of runs for each training epoch.

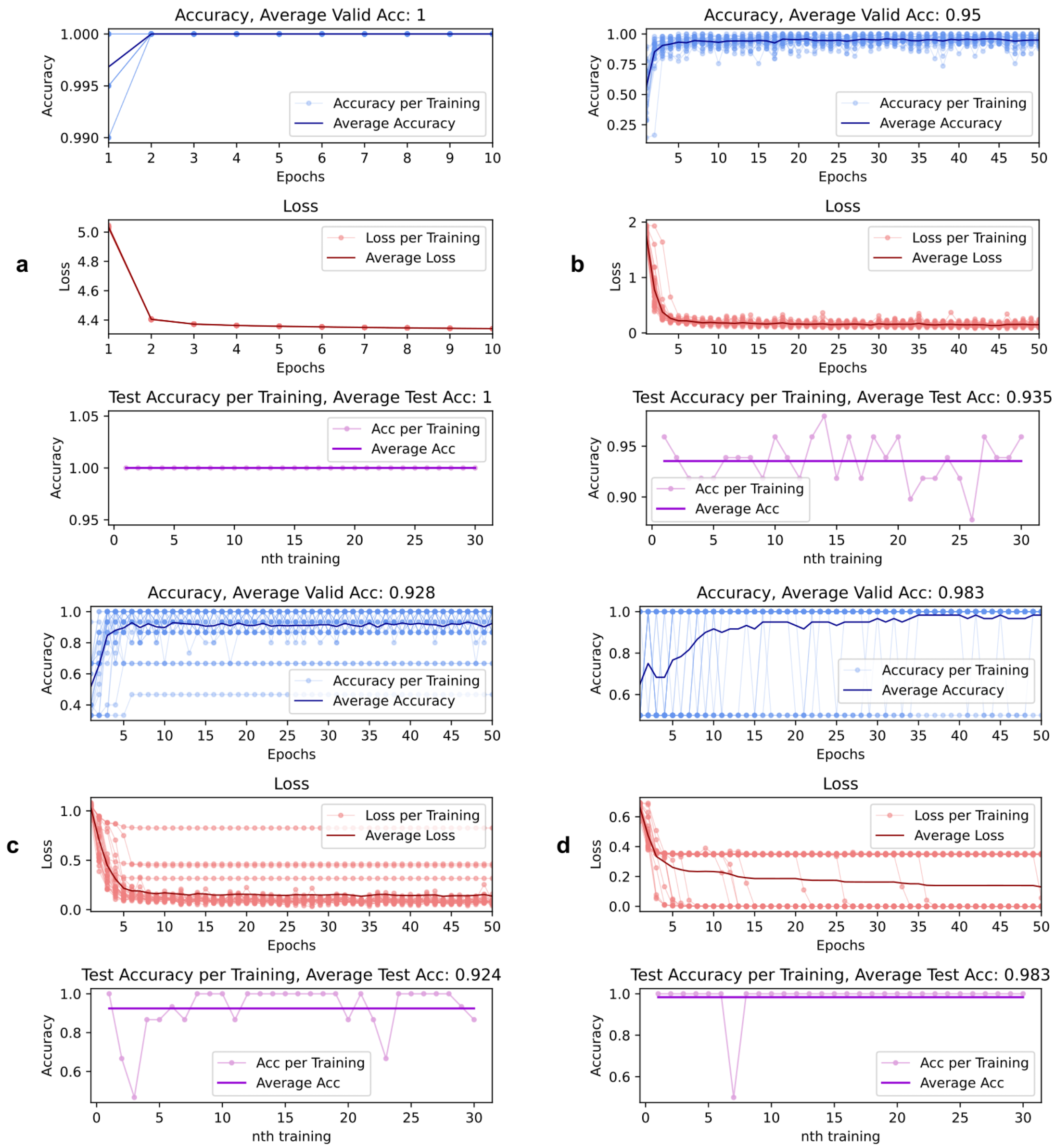

Fig. S5.

Performance of the digital networks. a, Binary vector classification. **b**, Vowel recognition. **c**, Iris plants classification. **d**, XOR task. In each panel, the top plots show validation accuracy versus training epochs (light blue: individual runs; dark blue: average across 30 runs, with the averaged validation accuracies reported in the titles). The middle plots show training loss versus epochs (dash-dotted red: individual runs; solid red: average). The bottom plots show test accuracy across 30 runs (dash-dotted lines: individual runs; solid line: average).

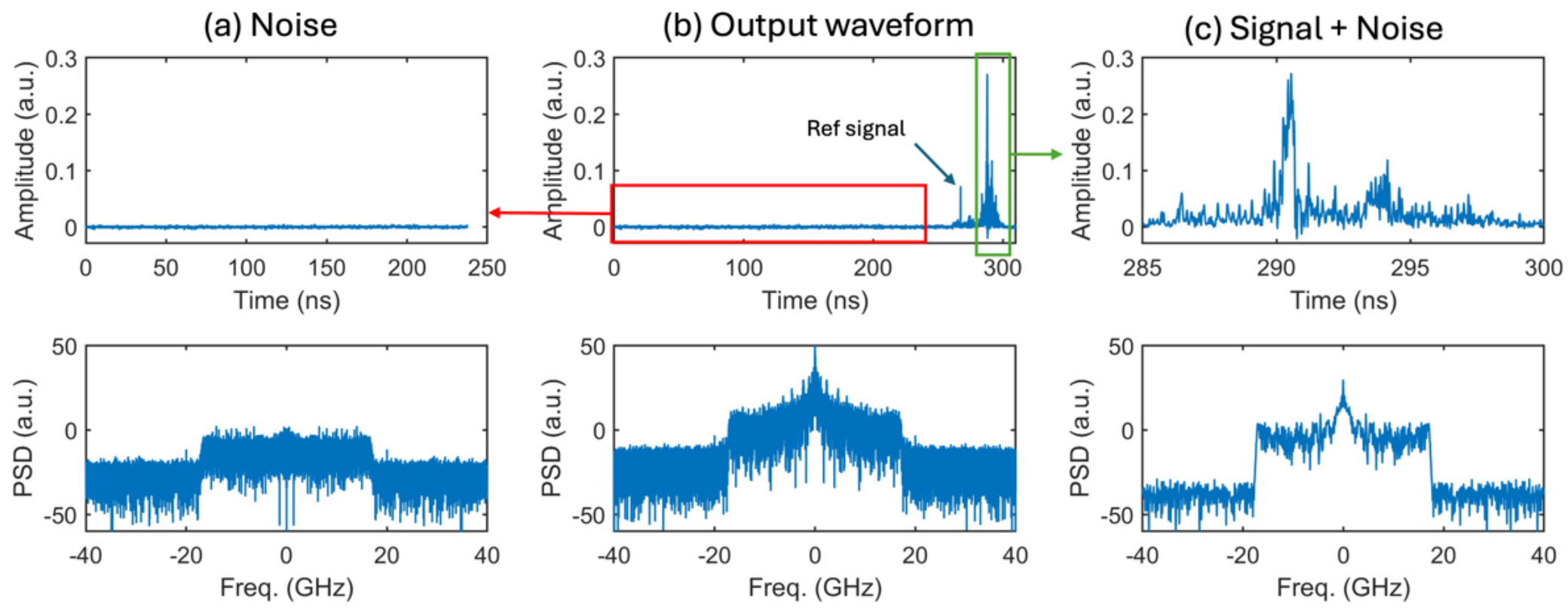

Fig. S6

Estimation of the SNR. a, Extracted temporal noise waveform and the power spectral density (PSD) after Fourier transform. **b,** 4th layer output waveform and spectrum of the 4-layer ONN. **c,** Extracted waveform including both signal and noise.

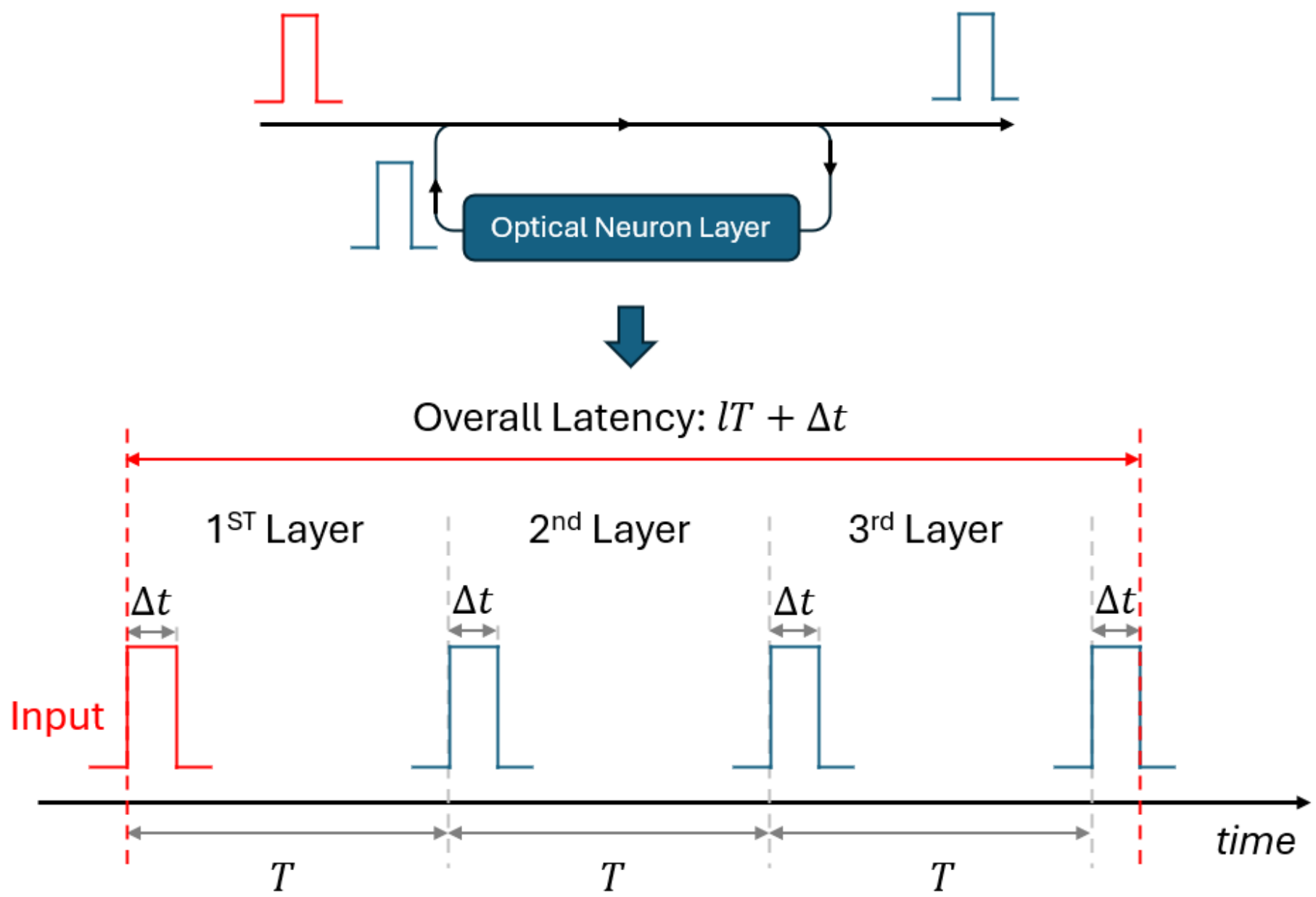

Fig. S7.

The overall latency of the ONN. Signal propagation through a three-layer Optical Neural Network (ONN). The input is shown as a red rectangular waveform, while the blue signals are the sequential outputs from each layer. For example, the second waveform from the left is the output from the first layer (also the input to the second layer). To prevent interference or overlapping between these waveforms, the roundtrip time (T) must be at least equal or longer than the waveform duration (Δt). For the three-layer network shown, this results in an overall latency of $3T + \Delta t$.

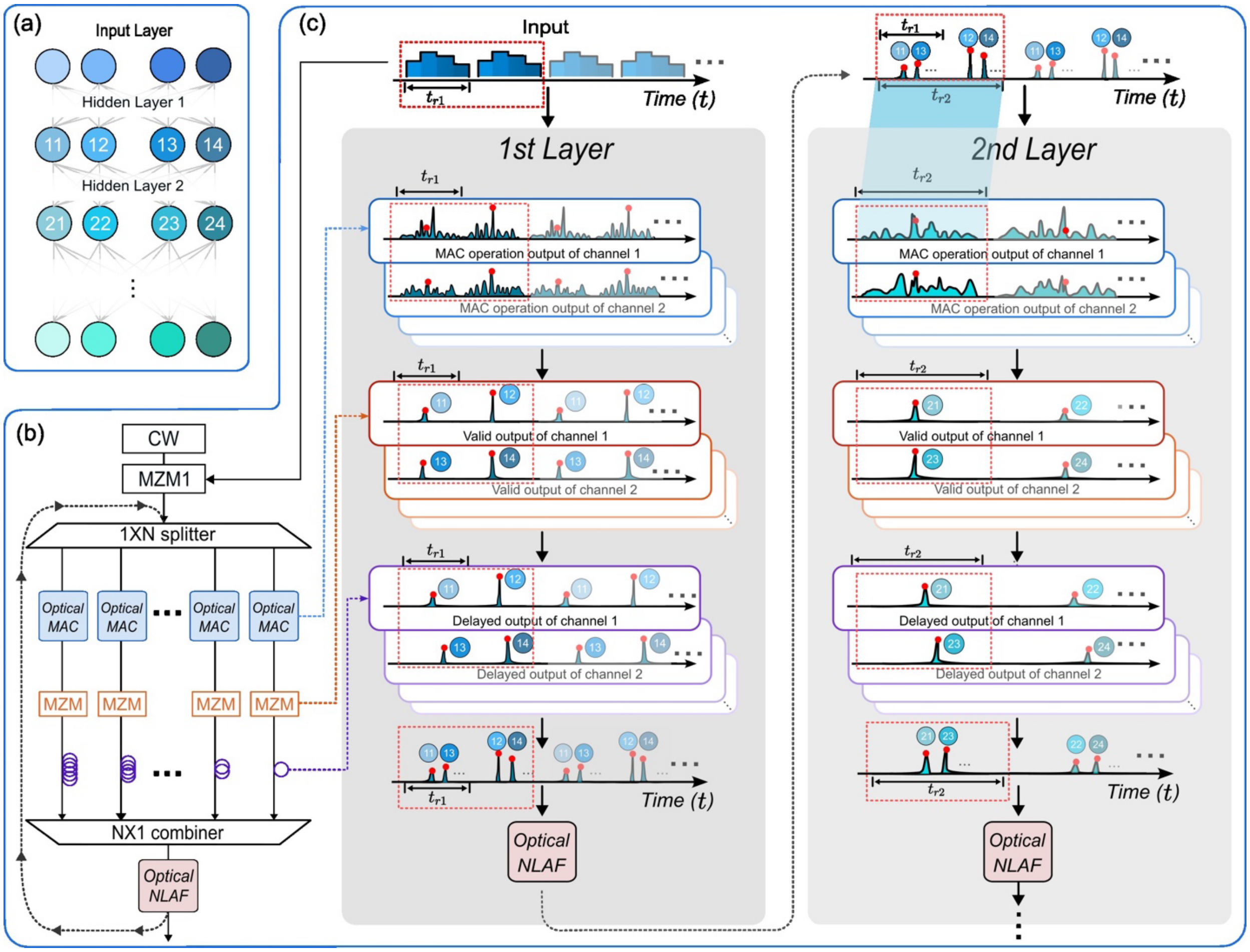

Fig. S8.

End-to-end optical neural network (ONN) based on hybrid time–space multiplexing. a, Schematic of a conventional deep neural network. **b**, Proposed architecture, where the input is encoded onto a temporal optical waveform and distributed into N parallel channels, each implementing an optical multiply–accumulate (MAC) operation (using a Mach-Zehnder modulator (MZM), a phase modulator, and a dispersive element). Valid outputs are selected, temporally aligned using delay lines, recombined, and processed by a shared optical nonlinear activation function (NLAF). **c**, Signal flow for a two-layer example with four neurons per layer, illustrating parallel MAC operations across N = 2 spatial channels and sequential generation of neuron outputs in the time domain.

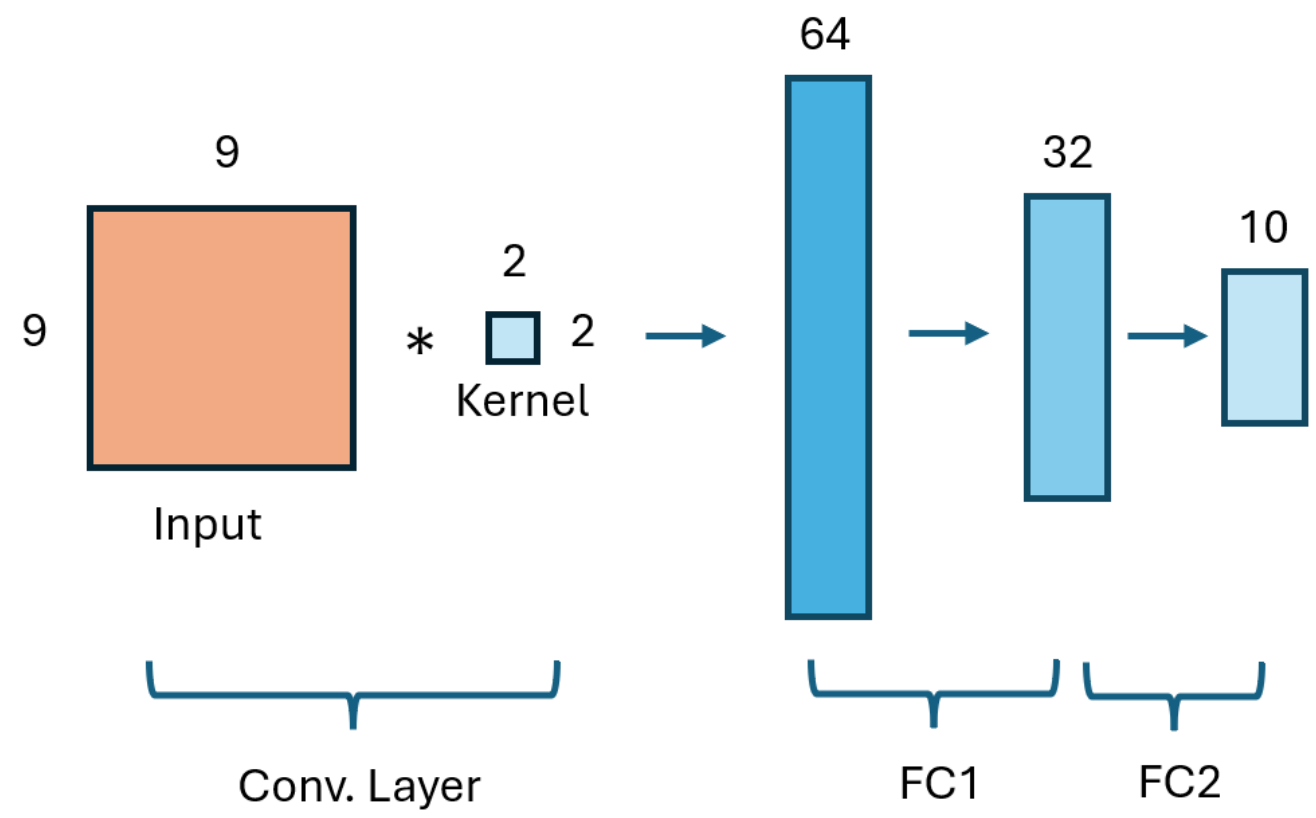

Fig. S9.
The architecture of the proposed CNN. We implement the *tanh* activation function in the convolutional (Conv.) layer and the fully connect (FC) layer 1.

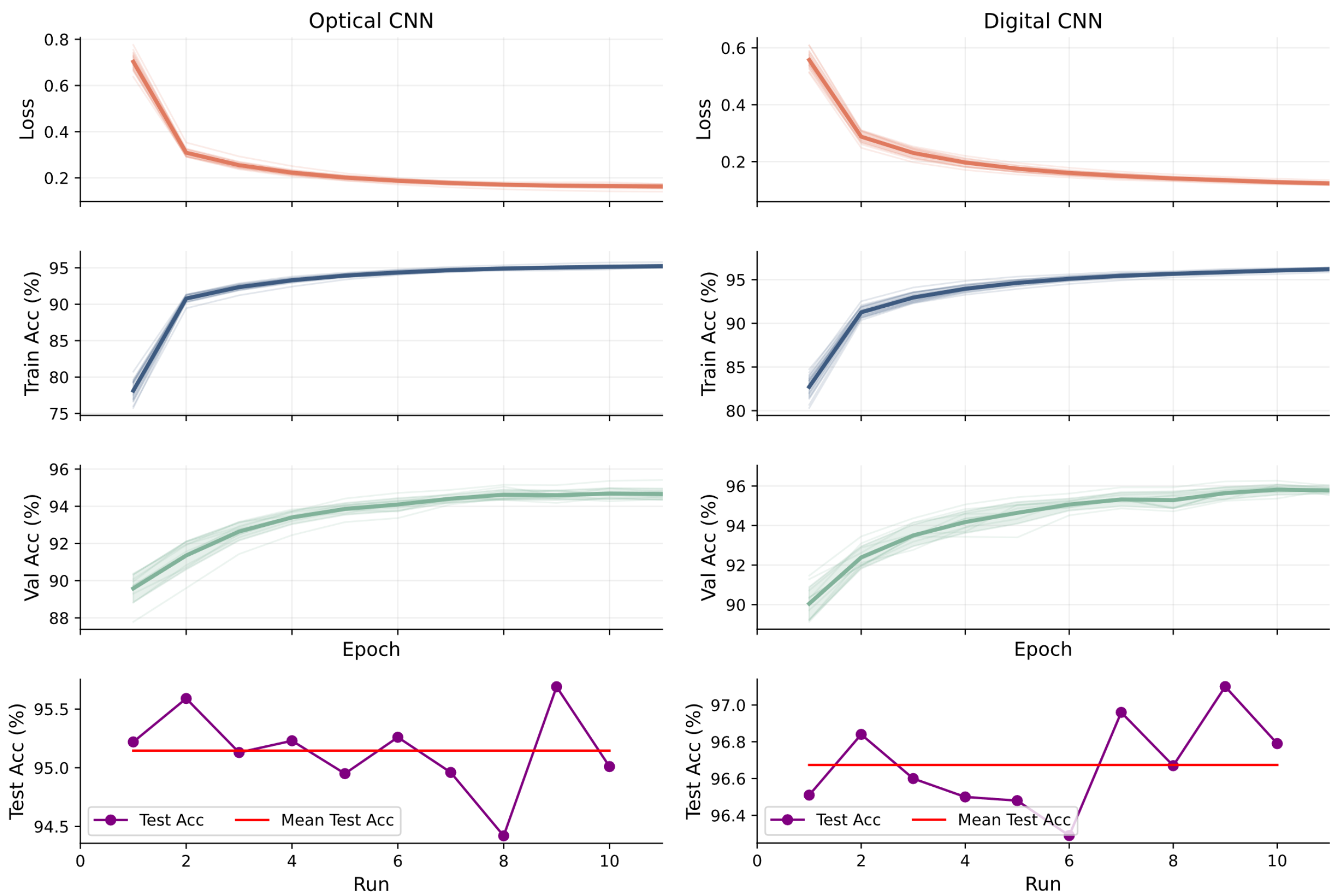

Fig. S10.

The simulation results of the proposed optical CNN (left) and the digital CNN (right). Top to bottom: training loss, training accuracy, validation accuracy, and the test accuracy for each run.

```python
gpu_info = !nvidia-smi
gpu_info = '\n'.join(gpu_info)
if gpu_info.find('failed') >= 0:
  print('Not connected to a GPU')
else:
  print(gpu_info)
```

```
Sat Sep  6 04:24:04 2025
+-----------------------------------------------------------------------------------------+
| NVIDIA-SMI 550.54.15              Driver Version: 550.54.15      CUDA Version: 12.4     |
|-----------------------------------------+------------------------+----------------------+
| GPU  Name                 Persistence-M | Bus-Id          Disp.A | Volatile Uncorr. ECC |
| Fan  Temp   Perf          Pwr:Usage/Cap |           Memory-Usage | GPU-Util  Compute M. |
|                                         |                        |               MIG M. |
|=========================================+========================+======================|
|   0  NVIDIA A100-SXM4-80GB          Off |   00000000:00:05.0 Off |                    0 |
| N/A   35C    P0             62W /  400W |     531MiB /  81920MiB |      0%      Default |
|                                         |                        |             Disabled |
+-----------------------------------------+------------------------+----------------------+

+-----------------------------------------------------------------------------------------+
| Processes:                                                                              |
|  GPU   GI   CI        PID   Type   Process name                              GPU Memory |
|        ID   ID                                                               Usage      |
|=========================================================================================|
+-----------------------------------------------------------------------------------------+
```

Fig. S11.

The static power consumption of the NVIDIA A100 GPU. Power consumption is marked in the red box.

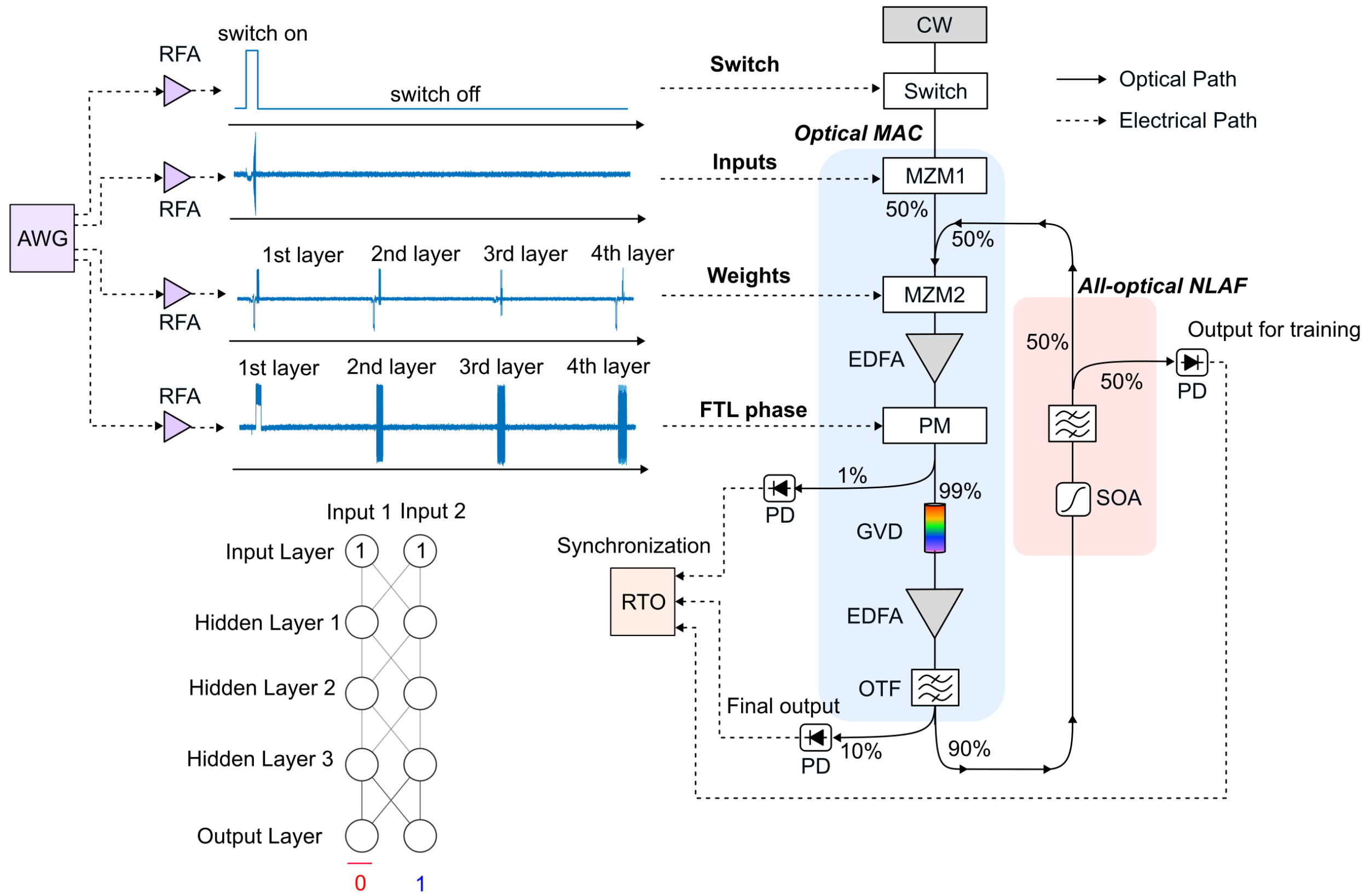

Fig. S12.

Experimental setup. A 92 GS/s arbitrary waveform generator (AWG) and four radio frequency amplifiers (RFAs) are used for data encoding. In this example, we design four temporal waveforms for optical switch, input encoding, weights encoding, and Fresnel time lens, successfully implementing a four-layer deep neural network (DNN) for XOR problem. Detailed waveforms are presented in Fig. S11. Specifically, input features are synchronized with weights and the Fresnel time lens (FTL) signals of the first hidden layer, while weights in the second hidden layer are matched with the returned signal. At the receiver end, three photodetectors (PDs) and a real-time oscilloscope (RTO) are used to measure the optical outputs. Note that the AWG and RTO are not involved in the inter-layer transmission.

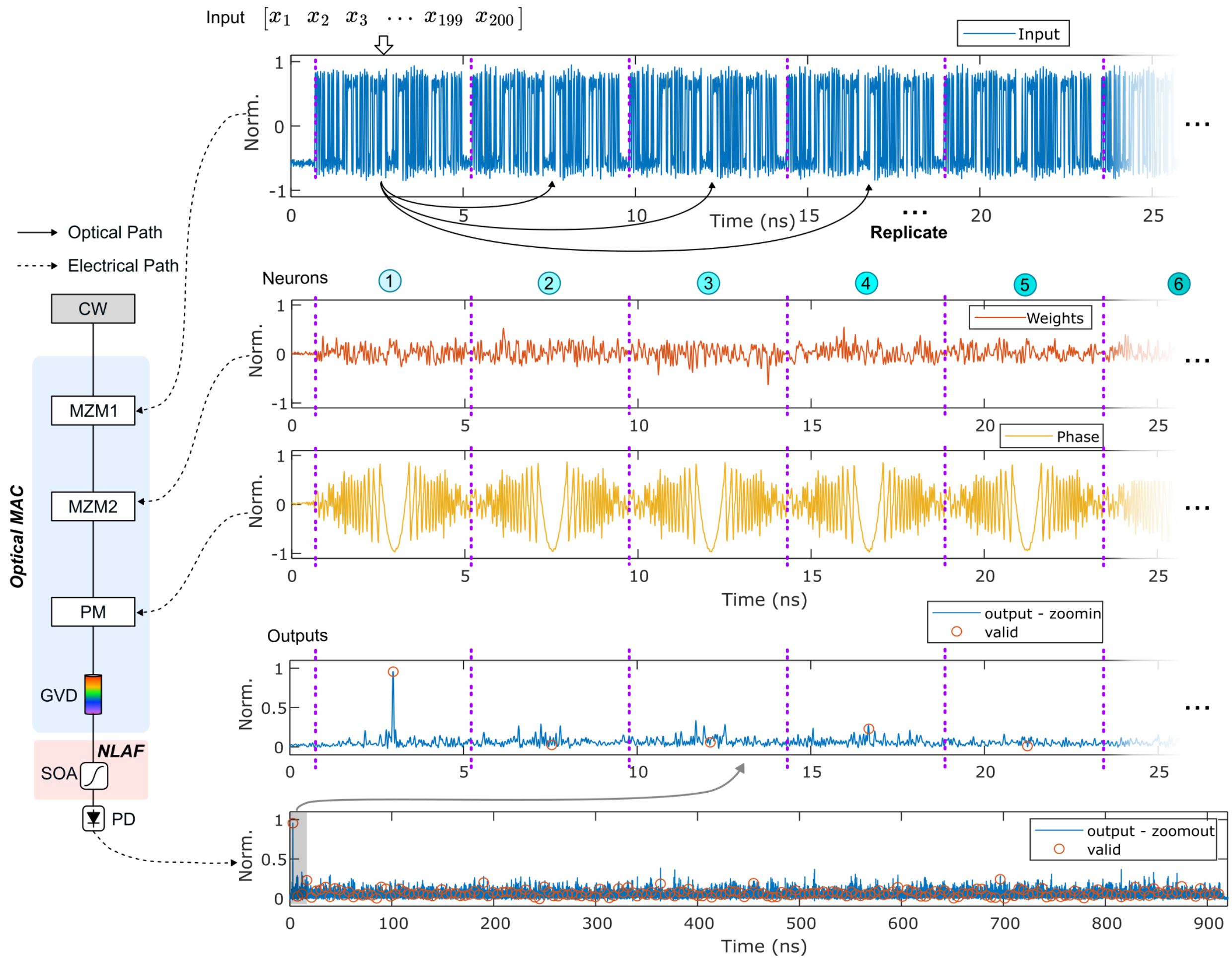

Fig. S13.
Experimental implementation of the single layer perceptron for the classification of 200 binary vectors.

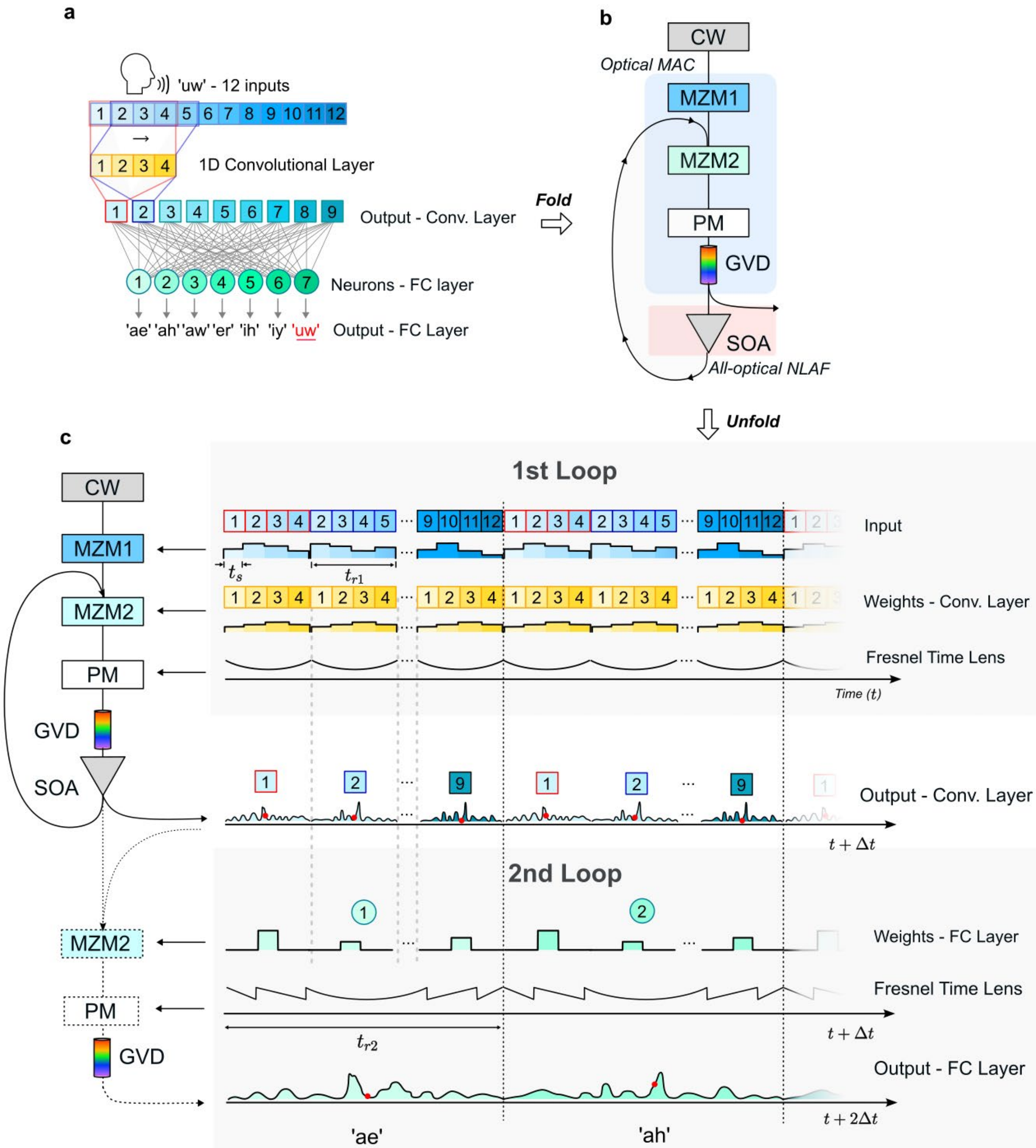

Fig. S14.

Conceptual illustration of the optical 1D CNN. a, Network topology for vowel recognition across seven classes. The convolutional kernel contains four weights, producing nine outputs that serve as inputs to the fully connected (FC) layer. **b**, Schematic of the experimental setup. **c**, Implementation of the 1D CNN in the optical domain.

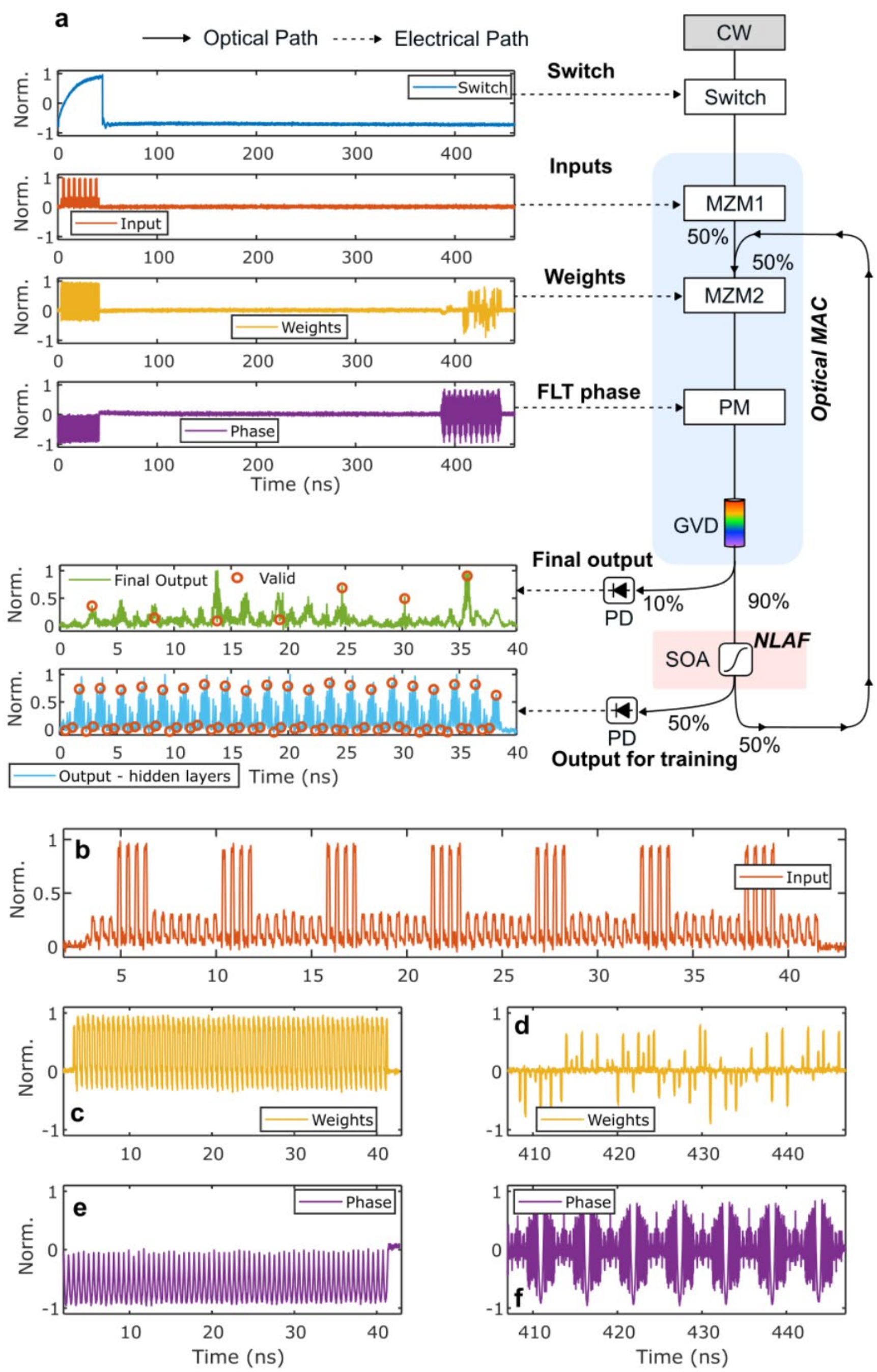

Fig. S15.

Experimental implementation of the one dimensional (1D) convolutional neural network (CNN) for the 7-class vowel recognition task. a, The overview of the experimental setup and waveforms. The top four waveforms represent the signals for the switch (blue), input data (red), weights (yellow), and the Fresnel time lens (FTL) phase profile (violet), while the bottom two are signals from the hidden (light blue) and output layers (green). **b**, The details of the input waveform. **c** and **d**, The weight waveforms of the convolutional layer and the fully connected (FC) layer, respectively. **e** and **f**, The phase modulation waveforms for the convolutional layer and the FC layer, respectively. Note that the switch signal (blue) is designed to compensate for the strong distortion introduced by the radio frequency amplifiers.

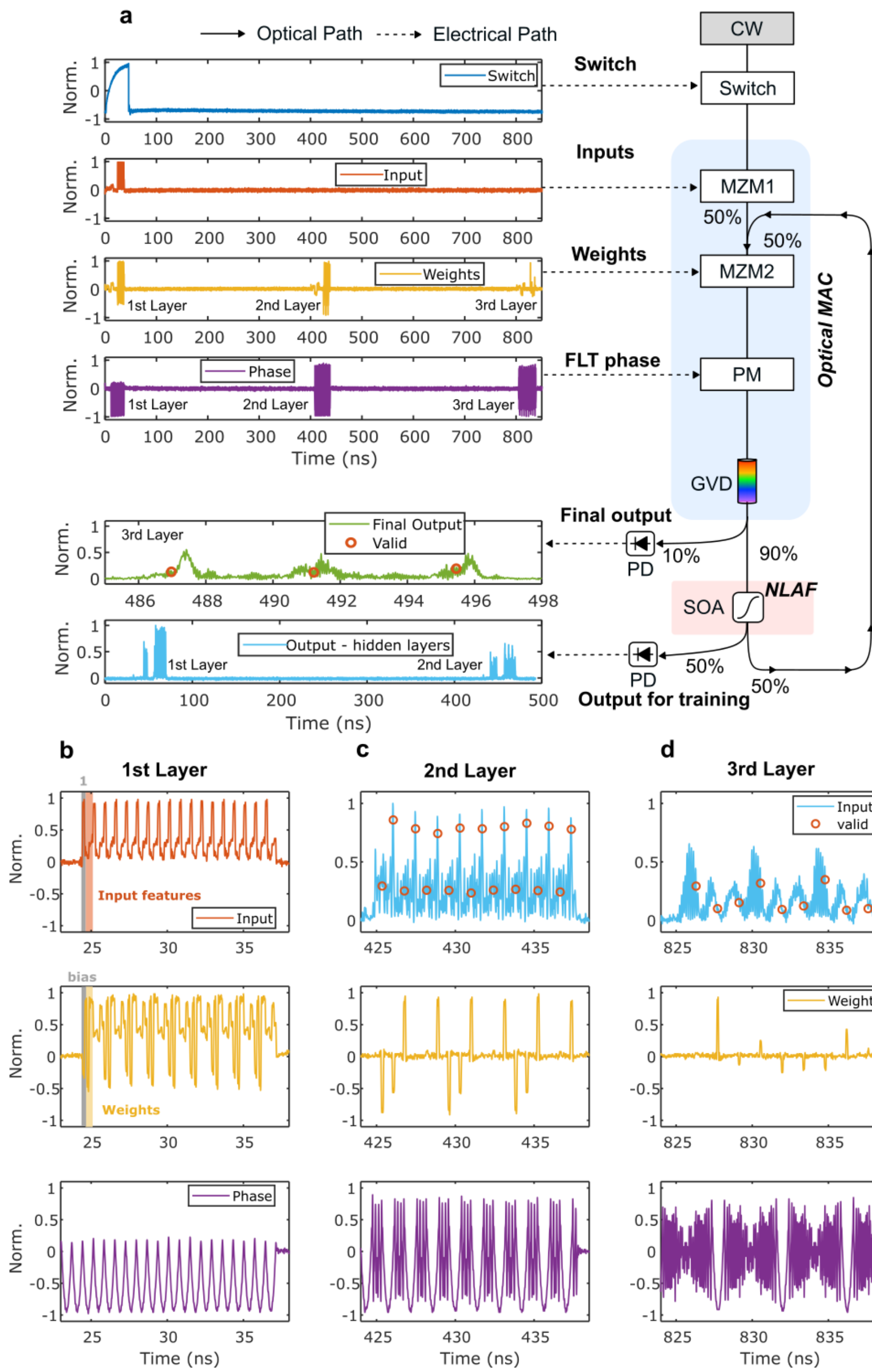

Fig. S16.

Experimental implementation of the 3-layer DNN for the Iris dataset. a, The overview of the experimental setup and waveforms. The top four waveforms represent the signals for the switch (blue), input data (red), weights (yellow), and the Fresnel time lens (FTL) phase profile (violet), while the bottom two waveforms are for the signals from the hidden (light blue) and output layers (green). **b, c,** and **d,** Input (red or light blue), weight (yellow), and phase modulation waveforms (violet) for the first, second, and third layer, respectively. Note that the switch signal (blue) is designed to compensate for the strong distortion introduced by the radio frequency amplifiers. t_{r1}~0.7 ns, t_{r2}~1.4 ns, t_{r3}~4.2 ns; t_{s1}~0.14 ns, t_{s2}~0.07 ns, t_{s3}~0.023 ns.

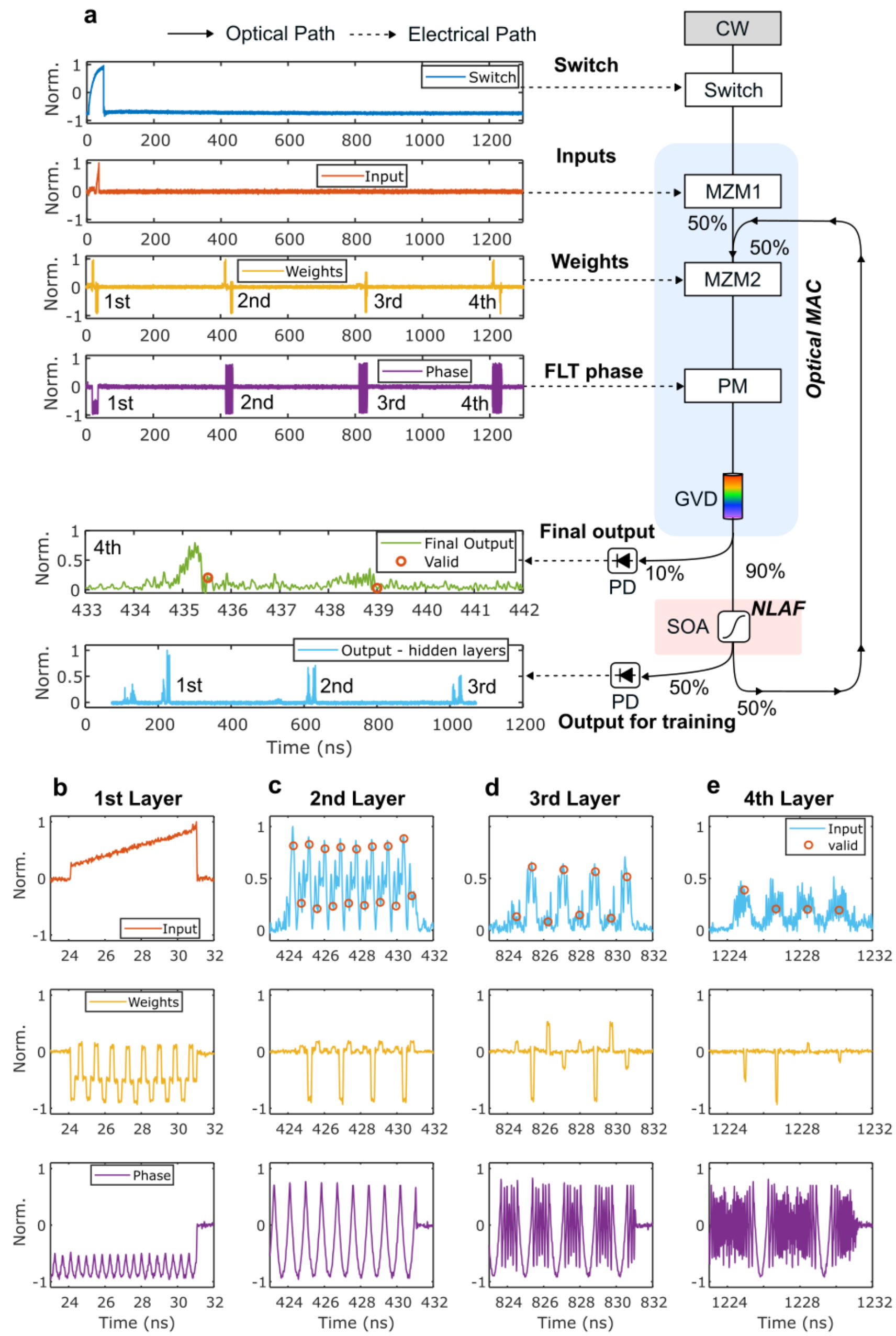

Fig. S17.

Experimental implementation of the 4-layer DNN for the XOR problem. a, The overview of the experimental setup and waveforms. The top four waveforms represent the signals for the switch (blue), input data (red), weights (yellow), and the Fresnel time lens (FTL) phase profile (violet), while the bottom two waveforms are for the output signals from the hidden (light blue) and output layers (green). **b, c, d,** and **e,** Input (red or light blue), weight (yellow), and phase modulation waveforms (violet) for the first, second, third, and fourth layer, respectively. Note that the switch signal (blue) and the input waveform (red) are designed to compensated for the strong distortion introduced by the radio frequency amplifiers. t_{r1}~0.43 ns, t_{r2}~0.87 ns, t_{r3}~1.74 ns, t_{r4}~3.5 ns; t_{s1}~0.22 ns, t_{s2}~0.11 ns, t_{s3}~0.055 ns, t_{s4}~0.0275 ns.

Table S1.

Data splitting*

Task	Classes	Features	Training dataset	Validation dataset	Test dataset
Binary vectors classification	200	200	400<br>(2 per class)	200<br>(1 per class)	200<br>(1 per class)
Vowel recognition	7	12	161<br>(23 per class)	49<br>(7 per class)	49<br>(7 per class)
Iris data	3	4	120<br>(40 per class)	15<br>(5 per class)	15<br>(5 per class)
XOR problem	2	2	20<br>(10 per class)	10<br>(5 per class)	10<br>(5 per class)

* The number of samples per dataset

Table S2.

Hyperparameters used for training digital ANNs

	Initial Learning Rate	Number of epochs
Binary vectors classification	0.001	10
Vowel recognition	0.01	50
Iris data		
XOR problem		

Table S3.

Comparison between latencies* (in nanoseconds)

*ANN topology**	SLP (200 vectors classification)	1D CNN (vowel recognition)	3-layer DNN (Iris dataset)	4-layer DNN (XOR problem)
Number of neurons in the output layer n_l	200	7	3	2
Number of layers l	1	2	3	4
Experiments				
Latency per roundtrip T	~400			
Temporal aperture of the output layer t_{rl}	~4.5	~5.4	~4.2	~3.5
Waveform duration per layer: $n_l t_{rl}$	~900	~37.8	~12.6	~7
Overall latency: $lT + n_l t_{rl}$	~1300	~837.8	~1212.6	~1607
With the optimization of the fiber loop length				
Estimated Overall latency: $(l+1)n_l t_{rl}$	~904.5*	~113,4	~50.4	~35
*Optimization with high-performance components (256 GS/s AWG and GVD ~510 ps/nm)**				
Estimated t_{rl}	N/A	~1.1	~0.98	~0.75
Estimated Waveform duration per layer: $n_l t_{rl}$	N/A	~7.7	~3	~1.5
Estimated Overall latency: $(l+1)n_l t_{rl}$	N/A	~.1	~12	~7.5
Electronic Processor (Digital processing latency per sample)				
Nvidia A100 GPU	~52,700	~108,400	~114,700	~143,000

* Latency here refers to the processing time per sample.
* The ONN topologies are the same as those reported in the main text.
* The minimum latency of the SLP is given by $n_l t_{rl}$(waveform duration) + t_{rl}(minimum physical latency).
* Integrated photonic devices are required to minimize the overall latency.

Table S4.

Estimation of the scalability

Task	Vowel recognition			Iris dataset			XOR problem		
ONN topology	1D CNN (without bias)			DNN (bias in the first layer)			DNN (without bias)		
	NN*	TA (ns)*	FS*	NN	TA (ns)	FS + 1 (bias)	NN	TA (ns)	FS
1st layer	9 (conv.)	~2.84	4	3	~3.2	4+1	2	~2	2
2nd layer	20	~25.56		3	~9.6		2	~4	
3rd layer	7	~511.2		3	~28.8		2	~8	
4th layer				3	~86.4		2	~16	
5th layer			N/A	3	~259.2	N/A	2	~32	N/A
6th layer	N/A	N/A					2	~64	
7th layer				N/A	N/A		2	~128	
8th layer							2	~256	

*NN: Number of neurons; TA: Temporal aperture; FS: Fan-in size.

Table S5

Comparison between ONN architectures and GPU

	Power consumption	Processing latency	Architecture	Reconfigurability
Ref. (*18*)	~ 3.75 W	~ 570 ps (3 layers, 9 neurons)	Stacked	×
Ref. (*19*)	~ 5 W (best case)	~ 410 ps (3 layers, 18 neurons)		
Ref. (*20*)	~ 10.57 W	~ 4.1 ns (4 layers, 30 neurons)		
NVIDA A100 GPU	~ 60 W (static)	~tens to hundreds of μs (estimated using the same architecture as for our work)	N.A.	✓
Our work	~ 6 W*	~ μ s (up to 200 neurons for SLP)	Recirculated loop	✓

* The power consumption of our work is roughly estimated using integrated devices.

Movie S1.

Real-time classification of the 200 random binary patterns. The test data is fed into the optical neural network sequentially from class 0 to class 199.

REFERENCES

1. Y. LeCun, Y. Bengio, G. Hinton, Deep learning. *Nature* **521**, 436–444 (2015).

2. Z. Li, X. Zhou, Z. Li, G. Peng, Y. Guo, L. Zhang, Packet header recognition utilizing an all-optical reservoir based on reinforcement-learning- optimized double-ring resonators. *IEEE J. Sel. Top. Quantum Electron.* **29**, 1–8 (2023).

3. V. Agarwal, P. Pareek, L. Singh, B. Balaji, P. K. Dakua, V. Chaurasia, Design and analysis of all optical header recognition system employing combination of carrier reservoir SOA and conventional SOA. *Opt. Quantum Electron.* **56**, 83 (2023).

4. T.-D. Do, M.-T. Duong, Q.-V. Dang, M.-H. Le, “Real-Time Self-Driving Car Navigation Using Deep Neural Network” in *2018 4th International Conference on Green Technology and Sustainable Development (GTSD)* (IEEE, 2018), pp. 7–12.

5. Z. Liu, A. Amini, S. Zhu, S. Karaman, S. Han, D. L. Rus, “Efficient and Robust LiDAR-Based End-to-End Navigation” in *2021 IEEE International Conference on Robotics and Automation (ICRA)* (IEEE, 2021), pp. 13247–13254.

6. N. Farmakidis, B. Dong, H. Bhaskaran, Integrated photonic neuromorphic computing: opportunities and challenges. *Nat. Rev. Electr. Eng.* **1**, 358–373 (2024).

7. D. Marković, A. Mizrahi, D. Querlioz, J. Grollier, Physics for neuromorphic computing. *Nat. Rev. Phys.* **2**, 499–510 (2020).

8. H. Zhou, J. Dong, J. Cheng, W. Dong, C. Huang, Y. Shen, Q. Zhang, M. Gu, C. Qian, H. Chen, Z. Ruan, X. Zhang, Photonic matrix multiplication lights up photonic accelerator and beyond. *Light Sci. Appl.* **11**, 30 (2022).

9. L. G. Wright, T. Onodera, M. M. Stein, T. Wang, D. T. Schachter, Z. Hu, P. L. McMahon, Deep physical neural networks trained with backpropagation. *Nature* **601**, 549–555 (2022).

10. X. Xu, M. Tan, B. Corcoran, J. Wu, A. Boes, T. G. Nguyen, S. T. Chu, B. E. Little, D. G. Hicks, R. Morandotti, A. Mitchell, D. J. Moss, 11 TOPS photonic convolutional accelerator for optical neural networks. *Nature* **589**, 44–51 (2021).

11. B. Dong, F. Brückerhoff-Plückelmann, L. Meyer, J. Dijkstra, I. Bente, D. Wendland, A. Varri, S. Aggarwal, N. Farmakidis, M. Wang, G. Yang, J. S. Lee, Y. He, E. Gooskens, D.-L. Kwong, P. Bienstman, W. H. P. Pernice, H. Bhaskaran, Partial coherence enhances parallelized photonic computing. *Nature* **632**, 55–62 (2024).

12. Z. Xu, T. Zhou, M. Ma, C. Deng, Q. Dai, L. Fang, Large-scale photonic chiplet Taichi empowers 160-TOPS/W artificial general intelligence. *Science* **384**, 202–209 (2024).

13. S. Chen, Y. Li, Y. Wang, H. Chen, A. Ozcan, Optical generative models. *Nature* **644**, 903–911 (2025).

14. J. Feldmann, N. Youngblood, M. Karpov, H. Gehring, X. Li, M. Stappers, M. Le Gallo, X. Fu, A. Lukashchuk, A. S. Raja, J. Liu, C. D. Wright, A. Sebastian, T. J. Kippenberg, W. H. P. Pernice, H. Bhaskaran, Parallel convolutional processing using an integrated photonic tensor core. *Nature* **589**, 52–58 (2021).

15. K. P. Kalinin, J. Gladrow, J. Chu, J. H. Clegg, D. Cletheroe, D. J. Kelly, B. Rahmani, G. Brennan, B. Canakci, F. Falck, M. Hansen, J. Kleewein, H. Kremer, G. O'Shea, L. Pickup, S. Rajmohan, A. Rowstron, V. Ruhle, L. Braine, S. Khedekar, N. G. Berloff, C. Gkantsidis, F. Parmigiani, H. Ballani, Analog optical computer for AI inference and combinatorial optimization. *Nature* **645**, 354–361 (2025).

16. F. Brückerhoff-Plückelmann, A. P. Ovvyan, A. Varri, H. Borras, B. Klein, L. Meyer, C. D. Wright, H. Bhaskaran, G. S. Syed, A. Sebastian, H. Fröning, W. Pernice, Probabilistic photonic computing for AI. *Nat. Comput. Sci.* **5**, 377–387 (2025).

17. C. Wang, Y. Cheng, Z. Xu, Q. Dai, L. Fang, Diffractive tensorized unit for million-TOPS general-purpose computing. *Nat. Photonics* **19**, 1078–1087 (2025).

18. F. Ashtiani, A. J. Geers, F. Aflatouni, An on-chip photonic deep neural network for image classification. *Nature* **606**, 501–506 (2022).

19. S. Bandyopadhyay, A. Sludds, S. Krastanov, R. Hamerly, N. Harris, D. Bunandar, M. Streshinsky, M. Hochberg, D. Englund, Single-chip photonic deep neural network with forward-only training. *Nat. Photonics* **18**, 1335–1343 (2024).

20. B. Wu, C. Huang, J. Zhang, H. Zhou, Y. Wang, J. Dong, X. Zhang, Scaling up for end-to-end on-chip photonic neural network inference. *Light Sci. Appl.* **14**, 328 (2025).

21. J. Feldmann, N. Youngblood, C. D. Wright, H. Bhaskaran, W. H. P. Pernice, All-optical spiking neurosynaptic networks with self-learning capabilities. *Nature* **569**, 208–214 (2019).

22. Y. Zuo, B. Li, Y. Zhao, Y. Jiang, Y.-C. Chen, P. Chen, G.-B. Jo, J. Liu, S. Du, All-optical neural network with nonlinear activation functions. *Optica* **6**, 1132–1137 (2019).

23. T. Wang, M. M. Sohoni, L. G. Wright, M. M. Stein, S.-Y. Ma, T. Onodera, M. G. Anderson, P. L. McMahon, Image sensing with multilayer nonlinear optical neural networks. *Nat. Photonics* **17**, 408–415 (2023).

24. J. Spall, X. Guo, A. I. Lvovsky, Hybrid training of optical neural networks. *Optica* **9**, 803–811 (2022).

25. L. De Marinis, A. Catania, P. Castoldi, G. Contestabile, P. Bruschi, M. Piotto, N. Andriolli, A codesigned integrated photonic electronic neuron. *IEEE J. Quantum Electron.* **58**, 1–10 (2022).

26. Z. Lin, B. J. Shastri, S. Yu, J. Song, Y. Zhu, A. Safarnejadian, W. Cai, Y. Lin, W. Ke, M. Hammood, T. Wang, M. Xu, Z. Zheng, M. Al-Qadasi, O. Esmaeeli, M. Rahim, G. Pakulski, J. Schmid, P. Barrios, W. Jiang, H. Morison, M. Mitchell, X. Guan, N. A. F. Jaeger, L. A. Rusch, S. Shekhar, W. Shi, S. Yu, X. Cai, L. Chrostowski, 120 GOPS Photonic tensor core in thin-film lithium niobate for inference and in situ training. *Nat. Commun.* **15**, 9081 (2024).

27. Z. Li, R. Gan, Z. Chen, Z. Deng, R. Gao, K. Chen, C. Guo, Y. Zhang, L. Liu, S. Yu, J. Liu, Scalable on-chip optoelectronic Ising machine utilizing thin-film lithium niobate photonics. *ACS Photonics* **11**, 1703–1714 (2024).

28. J. Azaña, X. Zhu, C. Rowe, B. Crockett, Optical time-mapped spectrograms (II): Fractional talbot designs. *J. Lightwave Technol.* **41**, 5284–5295 (2023).

29. J. M. Donohue, Fresnel time lens empowers quantum networks. *Nat. Photonics* **17**, 738–739 (2023).

30. F. Sośnicki, M. Mikołajczyk, A. Golestani, M. Karpiński, Interface between picosecond and nanosecond quantum light pulses. *Nat. Photonics* **17**, 761–766 (2023).

31. O. Destras, S. Le Beux, F. G. De Magalhães, G. Nicolescu, Survey on activation functions for optical neural networks. *ACM Comput Surv* **56**, 1–30 (2024).

32. J. Soni, N. Prabakar, H. Upadhyay, "Visualizing High-Dimensional Data Using t-Distributed Stochastic Neighbor Embedding Algorithm" in *Principles of Data Science*, H. R. Arabnia, K. Daimi, R. Stahlbock, C. Soviany, L. Heilig, K. Brüssau, Eds. (Springer International Publishing, Cham, 2020), pp. 189–206.

33. M. Romera, P. Talatchian, S. Tsunegi, F. Abreu Araujo, V. Cros, P. Bortolotti, J. Trastoy, K. Yakushiji, A. Fukushima, H. Kubota, S. Yuasa, M. Ernoult, D. Vodenicarevic, T. Hirtzlin, N. Locatelli, D. Querlioz, J. Grollier, Vowel recognition with four coupled spin-torque nano-oscillators. *Nature* **563**, 230–234 (2018).

34. P. Baldi, *Deep Learning in Science* (Cambridge Univ. Press, Cambridge, 2021).

35. R. A. Fisher, Iris, UCI Machine Learning Repository (1936); https://doi.org/10.24432/C56C76.

36. D. Masters, C. Luschi, Revisiting small batch training for deep neural networks. arXiv:1804.07612 [cs.LG] (2018).

37. N. S. Keskar, D. Mudigere, J. Nocedal, M. Smelyanskiy, P. T. P. Tang, On large-batch training for deep learning: Generalization gap and sharp minima. arXiv:1609.04836 [cs.LG] (2017).

38. L. Vanneschi, S. Silva, "Artificial Neural Networks" in *Lectures on Intelligent Systems*, L. Vanneschi, S. Silva, Eds. (Springer International Publishing, Cham, 2023), pp. 161–204.

39. G. Cong, S. Namiki, Photonic neural networks: Principles, progress, and prospects toward streaming processes. *J. Lightwave Technol.* **44**, 821–844 (2026).

40. S. Kaushal, A. Roberge, R. Kashyap, J. Azaña, Ultra-compact silicon photonics highly dispersive elements for low-latency signal processing. *Opt. Express* **31**, 3467–3478 (2023).

41. C. Wang, M. Zhang, X. Chen, M. Bertrand, A. Shams-Ansari, S. Chandrasekhar, P. Winzer, M. Lončar, Integrated lithium niobate electro-optic modulators operating at CMOS-compatible voltages. *Nature* **562**, 101–104 (2018).

42. Y. Liu, H. Zhang, J. Liu, L. Lu, J. Du, Y. Li, Z. He, J. Chen, L. Zhou, A. W. Poon, Parallel wavelength-division-multiplexed signal transmission and dispersion compensation enabled by soliton microcombs and microrings. *Nat. Commun.* **15**, 3645 (2024).

43. T. Matsumoto, T. Kurahashi, R. Konoike, K. Suzuki, K. Tanizawa, A. Uetake, K. Takabayashi, K. Ikeda, H. Kawashima, S. Akiyama, S. Sekiguchi, Hybrid-integration of SOA on silicon photonics platform based on flip-chip bonding. *J. Lightwave Technol.* **37**, 307–313 (2019).

44. G. Chen, Y. Yu, Y. Shi, N. Li, W. Luo, L. Cao, A. J. Danner, A.-Q. Liu, X. Zhang, High-speed photodetectors on silicon photonics platform for optical interconnect. *Laser Photonics Rev.* **16**, 2200117 (2022).

45. Chromatic Dispersion Emulator, Proximion. https://www.proximion.com/wp-content/uploads/2026/03/Datasheet-Chromatic-Dispersion-Emulator.pdf.

46. Keysight, M8199B 256 GSa/s Arbitrary Waveform Generator, Keysight. https://www.keysight.com/ca/en/product/M8199B/m8199b-256-gsa-s-arbitrary-waveform-generator.html.

47. A. Jha, C. Huang, P. R. Prucnal, Reconfigurable all-optical nonlinear activation functions for neuromorphic photonics. *Opt. Lett.* **45**, 4819–4822 (2020).

48. G. Mourgias-Alexandris, A. Tsakyridis, N. Passalis, A. Tefas, K. Vyrsokinos, N. Pleros, An all-optical neuron with sigmoid activation function. *Opt. Express* **27**, 9620–9630 (2019).

49. W. Yu, S. Zheng, Z. Zhao, B. Wang, W. Zhang, Reconfigurable low-threshold all-optical nonlinear activation functions based on an add-drop silicon microring resonator. *IEEE Photonics J.* **14**, 1–7 (2022).

50. T. O. Kehinde, W. A. Khan, S.-H. Chung, "Financial Market Forecasting using RNN, LSTM, BiLSTM, GRU and Transformer-Based Deep Learning Algorithms" in *Proceedings of the International Conference on Industrial Engineering and Operations Management* (IEOM Society International, Detroit, USA, 2023).

51. E. D. Black, An introduction to Pound–Drever–Hall laser frequency stabilization. *Am. J. Phys.* **69**, 79–87 (2001).

52. Erbium-Doped Fiber Amplifiers (EDFA), Fosco Connect. https://www.fiberoptics4sale.com/blogs/wave-optics/erbium-doped-fiber-amplifiers-edfas.

53. Low Power Amplifers. https://www.pritel.com/low_power_fa.html.

54. High Power CW Lasers – DenseLight Semiconductors. https://www.denselight.com/products/high-power-cw-lasers/.

55.1550 nm 3W High Power-Tunable Single Mode(SM) Fiber Laser module FLH-1550-35-SM-M21/M31. https://www.civillaser.com/index.php?main_page=product_info&products_id=3418.

56. EOSPACE, INC, EOSPACE, INC. https://www.eospace.com.

57. Semiconductor optical amplifiers (SOA), Innolume. https://www.innolume.com/innoproducts/semiconductor-optical-amplifiers-soa/.

58. D. Patel, "Design, Analysis, and Performance of a Silicon Photonic Traveling Wave Mach-Zehnder Modulator" (2014).

59. CrossEntropyLoss — PyTorch 2.8 documentation. https://docs.pytorch.org/docs/stable/generated/torch.nn.CrossEntropyLoss.html.

60. Adam — PyTorch 2.8 documentation. https://docs.pytorch.org/docs/stable/generated/torch.optim.Adam.html.

61. ReduceLROnPlateau — PyTorch 2.8 documentation. https://docs.pytorch.org/docs/stable/generated/torch.optim.lr_scheduler.ReduceLROnPlateau.html.

62. L. Prechelt, "Early Stopping - But When?" in *Neural Networks: Tricks of the Trade*, G. B. Orr, K.-R. Müller, Eds. (Springer, Berlin, Heidelberg, 1998), pp. 55–69.

63. P. Henderson, R. Islam, P. Bachman, J. Pineau, D. Precup, D. Meger, Deep reinforcement learning that matters. arXiv:1709.06560 [cs.LG] (2019).

64. M8196A 92 GSa/s Arbitrary Waveform Generator | Keysight. https://www.keysight.com/ca/en/product/M8196A/92-gsa-s-arbitrary-waveform-generators.html.

65. NVIDIA A100 GPUs Power the Modern Data Center, NVIDIA. https://www.nvidia.com/en-us/data-center/a100/.

66. C. Han, Q. Yang, J. Qin, Y. Zhou, Z. Zheng, Y. Zhang, H. Wang, Y. Sun, J. Lu, Y. Wang, Z. Ge, Y. Wu, L. Wang, Z. He, S. Yu, W. Hu, C. Peng, H. Shu, J. E. Bowers, X. Wang, Exploring 400 Gbps/λ and beyond with AI-accelerated silicon photonic slow-light technology. *Nat. Commun.* **16**, 6547 (2025).

67. H. Feng, T. Ge, X. Guo, B. Wang, Y. Zhang, Z. Chen, S. Zhu, K. Zhang, W. Sun, C. Huang, Y. Yuan, C. Wang, Integrated lithium niobate microwave photonic processing engine. *Nature* **627**, 80–87 (2024).

68. M. J. R. Heck, J. F. Bauters, M. L. Davenport, D. T. Spencer, J. E. Bowers, Ultra-low loss waveguide platform and its integration with silicon photonics. *Laser Photonics Rev.* **8**, 667–686 (2014).

69. T. Zhou, Y. Jiang, Z. Xu, Z. Xue, L. Fang, Hundred-layer photonic deep learning. *Nat. Commun.* **16**, 10382 (2025).

70. F. Ahmad, A. Mellati, A. Fernandez, A. Iyer, A. Fan, B. Reyes, C. Abidin, C. Nani, D. Albano, F. Solis, G. Minoia, G. Hatcher, H. Carrer, K. Kota, L. Wang, M. Bachu, M. Garampazzi, M. Hassanpourghadi, N. Fan, P. Prabha, R. Nguyen, S. Ho, T. Dusatko, T. Wu, W. Elsharkasy, Z. Sun, S. Jantzi, L. Tse, "18.3 An 8b 160GS/s 57GHz Bandwidth Time-Interleaved DAC and Driver-Based Transmitter with Adaptive Calibration for 800Gb/s Coherent Optical Applications in 5nm" in *2024 IEEE International Solid-State Circuits Conference (ISSCC)* (IEEE, 2024), vol. 67, pp. 342–344.

71. P. Caragiulo, O. E. Mattia, A. Arbabian, B. Murmann, "A Compact 14 GS/s 8-Bit Switched-Capacitor DAC in 16 nm FinFET CMOS" in *2020 IEEE Symposium on VLSI Circuits* (IEEE, 2020), pp. 1–2.

72. K. Li, D. J. Thomson, S. Liu, W. Zhang, W. Cao, C. G. Littlejohns, X. Yan, M. Ebert, M. Banakar, D. Tran, F. Meng, H. Du, G. T. Reed, An integrated CMOS–silicon photonics transmitter with a 112 gigabaud transmission and picojoule per bit energy efficiency. *Nat. Electron.* **6**, 910–921 (2023).

73. A. Giuglea, M. M. Khafaji, G. Belfiore, R. Henker, F. Ellinger, A low-distortion modulator driver with over 6.5-V_{pp} differential output swing and bandwidth above 60 GHz in a 130-nm SiGe BiCMOS technology. *IEEE Access* **10**, 78667–78676 (2022).

74. C-Band MSA EDFA Module, Low Power Consumption, Optilab. https://www.optilab.com/en-ca/products/c-band-msa-edfa-module-low-power-consumption.

75. C. Chen, F. Wei, X. Han, Q. Su, H. Pi, G. Xin, H. Wu, A. Stroganov, Y. Sun, W. Ren, X. Chen, Q. Ye, H. Cai, W. Chen, Hybrid integrated Si_3N_4 external cavity laser with high power and narrow linewidth. *Opt. Express* **31**, 26078–26091 (2023).

76. F. Iseini, A. Malignaggi, N. Pelagalli, A. Peczek, C. Carta, G. Kahmen, "44 GHz Bandwidth Optical Receiver Monolithically Integrated in a SiGe ePIC BiCMOS Technology" in *2025 IEEE 24th Topical Meeting on Silicon Monolithic Integrated Circuits in RF Systems (SiRF)* (IEEE, 2025), pp. 12–14.

77. L. Kull, D. Luu, C. Menolfi, M. Brändli, P. A. Francese, T. Morf, M. Kossel, A. Cevrero, I. Ozkaya, T. Toifl, A 24–72-GS/s 8-b time-interleaved SAR ADC With 2.0–3.3-pJ/Conversion and >30 dB SNDR at Nyquist in 14-nm CMOS FinFET. *IEEE J. Solid State Circuits* **53**, 3508–3516 (2018).
